# Effect on preoperative anxiety of a personalized three-dimensional kidney model prior to nephron-sparing surgery for renal tumor: study protocol for a randomized controlled trial (Rein 3D Print-Anxiety – UroCCR 113)

**DOI:** 10.1371/journal.pone.0321747

**Published:** 2025-04-24

**Authors:** Alice Pitout, Gaëlle Margue, Joffrey Sarrazin, Laura Richert, Hugo Larribère, Sarah Masanet, Thibaut Waeckel, Pierre Bigot, Romain Boissier, Bastien Parier, Stéphane De Vergie, Manon Jaffredo, Solène Ricard, Hélène Hoarau, Marthe-Aline Jutand, Matthieu Faessel, Jocelyn Sabatier, Jean-Christophe Bernhard

**Affiliations:** 1 Department of Urology, University Hospital, Bordeaux, France; 2 Emerging team - I.Care (Integrated research & innovation program for kidney cancer) - Inserm, UMR1312, BRIC, Bordeaux Institute of Oncology, Bordeaux University, Bordeaux, France; 3 Department of Additive Fabrication Engineering, TechnoShop Coh@bit, Bordeaux University Technological Institute, Gradignan, France; 4 Medical Information Department, University Hospital, Bordeaux cedex, France; 5 Culture et Diffusion des Savoirs EA-7440, Bordeaux University, Bordeaux, France; 6 Department of Urology, University Hospital, Caen, France; 7 Department of Urology, University Hospital, Angers, France; 8 Aix Marseille University, Department of Urology and Transplantation, University hospital La Conception, AP-HM, Marseille, France; 9 Department of Urology, Hôpital Bicêtre, AP-HP, Paris, France; 10 Department of Urology, University Hospital, Nantes, France; 11 Culture et Diffusion des Savoirs EA-7440, Bordeaux University, Bordeaux, France; 12 University of Bordeaux, Teachning Hospital of Bordeaux (CHU- URISH), Bordeaux, France; PLOS: Public Library of Science, UNITED KINGDOM OF GREAT BRITAIN AND NORTHERN IRELAND

## Abstract

**Background:**

The announcement of a diagnosis can be a source of anxiety for patients. Managing this anxiety is a major challenge, in terms of quality of life but also for the use of anxiolytic and analgesic therapies. The use of 3D modeling technology in partial nephrectomy surgery has proved its worth as a surgical aid but it could also help patients to manage their own care, by reducing their anxiety and increasing their understanding of the disease and its treatment. We aim to test this hypothesis with a prospective multicenter trial.

**Methods:**

R3DP-A (Rein 3D – Anxiety) is an unblinded, multicenter, randomized, prospective, superiority-controlled trial. Participants are patients with kidney tumors treated by robot-assisted partial laparoscopic nephrectomy. The 234 patients (78x3 groups) from 6 French centers will undergo a pre-operative consultation dedicated to a personalized explanation of the surgical management and its risks. They will be randomized into three (1:1:1) groups corresponding to three types of support for consultation: use of a virtual 3D model of the kidney and its tumor; a printed 3D model; or the standard information sheet from the French Association of Urology (control group). Several self-questionnaires will be sent by the UroConnect® application and completed at different times during the study. The primary endpoint will be pre-operative anxiety (STAI-state questionnaire completed the day before surgery D-1). Secondary endpoints will be changes in anxiety levels between the pre-operative and post-operative consultations (between inclusion and D15 post-op), changes in health literacy and quality of life (HLSEU-Q16 and EQ-5D-5L questionnaires at inclusion and D15), feelings of understanding of the disease and its treatment at pre-operative period (Wake questionnaire at D-1), and consultation times.

**Discussion:**

We aim to highlight a benefit of using a personalized 3D model on the anxiety level of patients undergoing partial nephrectomy surgery, as well as on their level of understanding of their pathology and its surgical treatment. The use of these models could be incorporated into current practice to improve patient experience throughout care.

## Introduction

The announcement of a cancer diagnosis is a source of anxiety and has psychological repercussions for the patient. This anxiety may be heightened by the explanation of surgery, which can be complex. In the case of kidney cancer, limited literature is available but seems to show moderate to severe psychological distress [1]. Thus, the patient’s perception of his pathology and its therapeutic management has a direct impact on his quality of life. Current treatments for pre-operative anxiety rely mainly on anxiolytics drugs [2]. The reduction in analgesics or anxiolytics promulgated by ERAS (Enhanced Recovery After Surgery) protocols may therefore be impacted by this state of anxiety. Improving pre-operative anxiety therefore seems to be a therapeutic challenge and is currently underdeveloped.

Over the past ten years, 3D modeling of kidneys and their tumors prior to partial nephrectomy has become an increasingly popular technological aid, both for educational purposes and for preoperative planning and intraoperative guidance in virtual reality [3]. The 3D-IGRAPN (3D Image-Guided Robot-Assisted Partial Nephrectomy) has shown improvements in terms of trifecta [4]. In addition, the use of 3D models during pre-operative consultations appears to improve patients’ understanding of renal and tumor anatomy, the surgical risks inherent in their own tumor (vascular or urinary lesions), and damage to renal volume [5–7]. These improvements appear to be clinically beneficial in terms of consultation time, compliance with care, and complications [8].

Few studies have examined the psychological beneficial impact of using a patient-specific model during the pre-operative consultation. These are retrospective, and often use non-specific Likert-type questionnaires. Finally, the specificities associated with the use of a printed or digital 3D model have rarely been analyzed [9].

We therefore aim to study the integration of a patient-specific 3D model during a pre-operative visit to patients diagnosed with kidney tumors before partial nephrectomy, to improve their experience in care, in a prospective multicenter trial.

## Materials and methods

**Fig 1:**
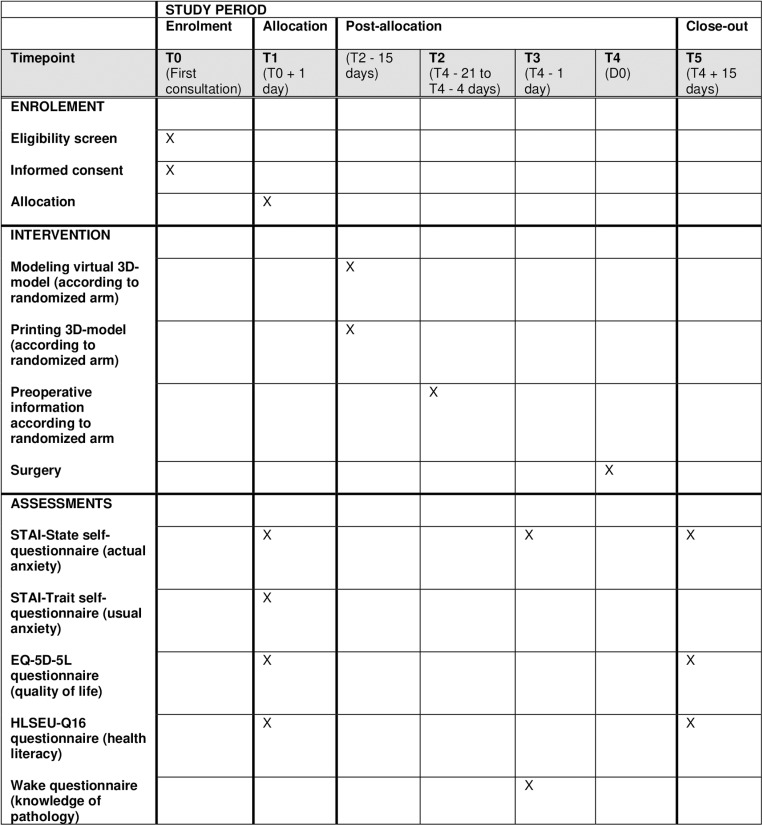
SPIRIT schedule.

**Fig 2:**
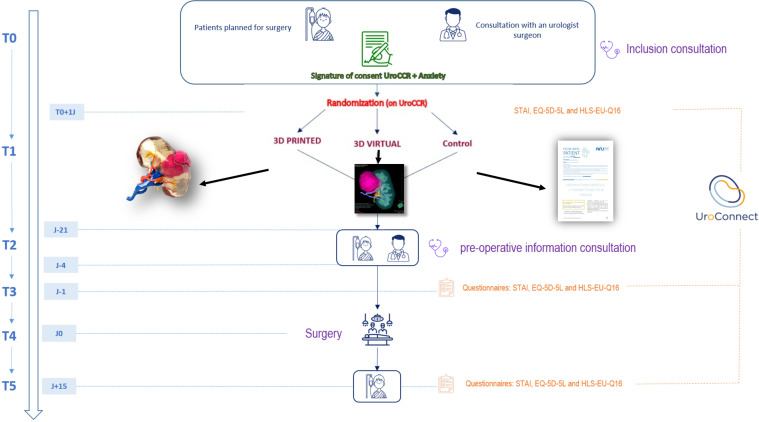
Study design.

We are planning a prospective unblinded multicenter superiority trial randomized in 3 parallel groups (1:1:1) according to the use of a digital 3D model, a printed 3D model, or the standard patient information sheet from the French Association of Urology as an information support during the preoperative consultation. We hypothesize that the use of a personalized model helps reduce perioperative anxiety, measured by self-questionnaires. The timeline is represented by SPIRIT schedule in Fig 1. The study design is presented in Fig 2.

### Study population and setting

The study will be carried out in 6 French centers of the UroCCR network (www.uroccr.fr) [10]: Bordeaux University Hospital, Kremlin-Bicêtre Paris University Hospital, Angers University Hospital, Caen University Hospital, Nantes University Hospital, Marseille University Hospital. The UroCCR network is a multi-center collaborative network of 53 centers in France, dedicated to collecting clinical, biological and follow-up data on patients undergoing kidney cancer surgery. With more than 18,500 patients, it currently represents one of the largest active cohorts of kidney cancer patients in the world. Patients will be recruited during diagnostic consultations of a kidney tumor before being scheduled for partial nephrectomy. The first inclusion took place on 19/09/2023. The end of inclusions is scheduled for September 2025 (24 months of inclusion). Data collection will be completed 4 months later, and results expected 6 months later. The protocol is in accordance with the Declaration of Helsinki. This research has been approved by the French Data Protection Committee. All participants will provide written informed consent. Patients can withdraw their consent at any time. He or she will benefit from the resumption of standard care, with no further consequences. Trial is registered with the reference NCT06035211 [ClinicalTrials.gov] [registered on 5 September 2023]. UroCCR network is registered with the reference NCT 03293563.

The inclusion and exclusion criteria are summarized in Table 1. Participation in the study will be offered to all consecutive patients meeting the eligibility criteria during the inclusion period.

**Table 1.** Inclusion and exclusion criteria.

Inclusion criteria	Exclusion criteria
Male and female aged of 18 and over	Language difficulties in French
Scheduled for robotic-assisted partial nephrectomy for a unilateral renal tumor, or first surgery for a bilateral tumor	Absence of a preoperative CT scan to produce the 3D model
Affiliation to or beneficiary of the French social security	Person under trusteeship, curatorship, or legal guardianship
Free, informed, and written consent signed by the patient and the investigating physician (at the latest on the day of inclusion and before any examination required by the research)	Refusal of consent or participation in the UroCCR project and the R3DP-A ancillary trial

*R3DP-A: Rein 3D – Anxiety*

All patients will sign an informed consent prior to inclusion in UroCCR and in the R3DP-A (Rein 3D - Anxiety) study. After inclusion, data will be anonymized and collected in the UroCCR database.

### Procedure

**Fig 3:**
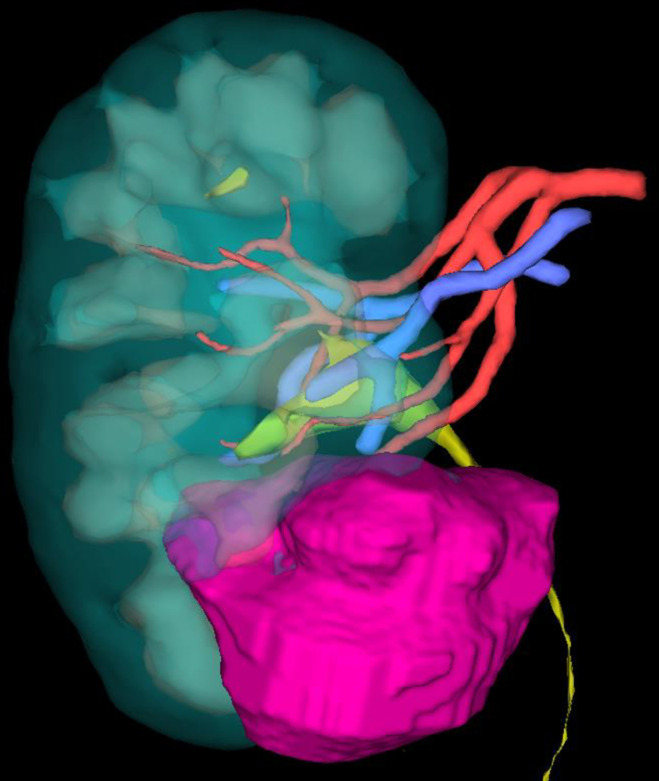
3D virtual model.

**Fig 4:**
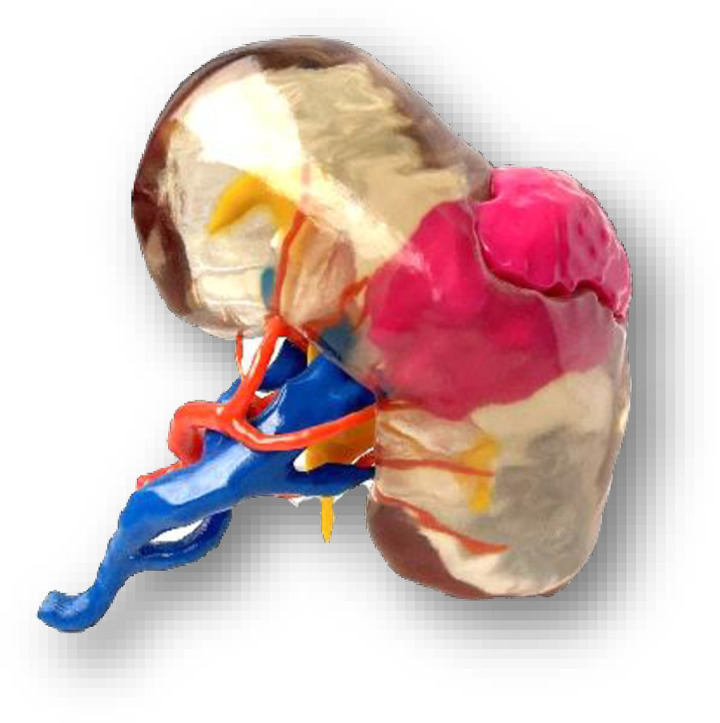
3D printed model.

Each patient will be randomly assigned to one of the three parallel groups (1:1:1) at inclusion: virtual 3D model (group 1) (Fig 3), printed 3D model (group 2) (Fig 4), or the patient information sheet from the French Urology Association only (group 3).

In Group 1, a preoperative virtual 3D model of the kidney and the tumor (digital twin) will be produced by the surgical team using the software Synapse 3D (Fujifilm). This model will be presented to the patient during a dedicated consultation between D-21 and D-4 before surgery. This consultation allows to present the surgery’s specificities linked to tumor characteristics and to answer the patient’s questions using this didactic model.

In the same way, a patient specific 3D printed model will be presented to Group 2. These models are based on the 3D digital model and printed using the Stratasys J750 printer (physical twin). It can be manipulated by the patient during this dedicated consultation.

In group 3, the control group, no 3D model will be presented to the patient. The consultation will be conducted using the patient information sheet from the French Urology Association, recommended in standard practice [11].

### Outcomes

The primary endpoint is the mean preoperative anxiety score. It will be measured one day before surgery by STAI-state self-questionnaire [12]. The STAI-state score is a self-administered questionnaire assessing short-term anxiety. Available in French [13], it consists of 20 items assessed by a 4-point Likert scale, rated from 20 (low anxiety) to 80 (high anxiety). Completion takes around 5–10 minutes.

Secondary endpoints are:

- Change in mean anxiety score, measured by STAI-state self-questionnaire and adjusted to STAI-trait score. For this purpose, the STAI-trait questionnaire will be completed at inclusion, to assess feelings of apprehension, tension, nervousness and worry usually experienced by the subject, one day before surgery and at 15 days after surgery.
- Evolution of the mean HLSEU-Q16 health literacy questionnaire score [14], French version [15], between inclusion and 15 days post-operative, which assesses 4 skills (accessing, understanding, evaluating, and applying health information). It consists of 16 items assessed by a 4-point Likert scale.
- Average pre-operative score for understanding the disease and the chosen treatment (Wake questionnaire [9]) measured one day before surgery.
- Evolution of the mean EQ-5D-5L score [16,17], a quality-of-life score in 5 dimensions (mobility, personal autonomy, impact on daily activities, pain and discomfort, anxiety, and depression), measured at inclusion and 15 days after surgery.
- Average duration of pre-operative consultation.

All questionnaires will be sent using the UroConnect® by Resilience application [18], a remote monitoring application also routinely used in our nurses-led ERAS and ambulatory perioperative pathways of care, or in paper format for patients without internet access. The application will alert the participant according to the research period to complete the appropriate questionnaire.

### Randomization and blinding

Randomization will be carried out on the UroCCR platform immediately after the inclusion visit and stratified by center. Once the patient has been included, and just before sending the questionnaires, the investigator logs on to the UroCCR.fr website using their confidential codes. Once the patient’s eligibility has been validated, the interface immediately communicates the participant’s unique number and the result of the randomization to the investigator. The randomization list is drawn up by the statistician at Bordeaux University Hospital’s Methodology and Data Management Center before the research begins. The study is unblinded, but patients will not be informed of their randomization group until the dedicated visit.

### Pre‐ and post‐operative follow‐up

Participants in the study will therefore receive an inclusion consultation (T0), corresponding to standard care. The STAI-trait, STAI-state, EQ5D-5L, and HLS-EU-Q16 questionnaires will be administered using the UroConnect® application the day after inclusion (T1). The patient will then be randomized to one of the three groups allowing the surgical team to produce the virtual or the printed 3D model. An additional consultation will be carried out between D-21 and D-4 before surgery (T2), to explain the surgical procedure and answer any questions the patient may have. In groups 1 and 2, this information will be based on the 3D model (virtual or printed). In the control group, only the standard information sheet of the French Urology Association will be used. The STAI-state and Wake questionnaires will be completed the day before surgery (T3). Surgery will be performed without changing the surgeon’s practice (D0, T4). The final STAI-state, EQ5D-5L, and HLS-EU-Q16 questionnaires will be completed on post-operative day 15 (T5). The patient will attend a post-operative consultation (between 1 and 3 months after surgery) as part of the usual follow-up, which is not part of the research.

### Calculation of the study size

We assume that the mean level of pre-operative anxiety assessed using STAI-state is 55 (based on the Bordeaux University Hospital pilot study [7]), and that a clinically significant difference is a 5-point reduction in the score. With a common standard deviation of 10, a static power of 80% and a bilateral type I error of 2.5% (2 comparisons with the control group in a 3-arm trial, Bonferroni correction), we need to include 78 patients per group, for a total of 234 patients.

The inclusion period will last 24 months, with each patient participating for 2–4 months, for a total research duration of 26–28 months.

### Data analysis

The primary endpoint will be analyzed on an intention-to-treat basis, followed by a per-protocol analysis. A descriptive analysis will be performed for each group, followed by comparisons of groups 1 and 2 with the control group, with and without adjustment for center and a Bonferonni adjustment. The tests used will be chi2 or corrected chi2 tests, or Fisher’s exact test, or Student’s t test, depending on the nature of the variable. Logistic regression and linear regression models will be applied to estimate differences between the randomization arms on the preoperative STAI-state score by adjusting for center, STAI-state score at inclusion, STAI-trait score at inclusion and including appropriate interaction terms. A secondary analysis will be performed on the per-protocol population. Statistical tests are performed with SAS software.

Missing data for questionnaires will be managed according to the score manual, and by multiple imputation if the overall score is missing or cannot be calculated. All patients who die, are lost to follow-up, or drop out will be included in the intention-to-treat analysis.

### Trial status

The protocol number is ID-RCB 2023-A00146-39, version no. 1 from 19/08/2024. Recruitment began in September 2023. Inclusion deadline is scheduled September 2025. Trial is registered with the reference NCT06035211 [ClinicalTrials.gov] [registered on 5 September 2023]. UroCCR network is registred with the reference NCT 03293563.

## Discussion

In this prospective, multicentric, randomized controlled trial, we aim to determine whether using personalized 3D kidney models (virtual or printed) during pre-operative consultations can reduce anxiety and improve understanding for patients undergoing robot-assisted partial nephrectomy, compared to standard information sheets. If effective, these models could enhance patient care and experience. Next, a prospective trial will be carried out to compare the use of the specific printed model with that of a generic printed model, to consider the use of a reduced number of premade models. This will assess the impact of the specificity of the model on the patient’s experience.

## Supporting information

S1 File Anxiety French protocol. (PDF)

## Abbreviations

3D-IGRAPN: 3D-Image Guided Robot Assisted Partial Nephrectomy
ERAS: enhanced recovery after surgery
R3DP-A: Rein 3D - Anxiety
