# Supplementary material for: Effect on preoperative anxiety of a personalized three-dimensional kidney model prior to nephron-sparing surgery for renal tumor: study protocol for a randomized controlled trial (Rein 3D Print-Anxiety – UroCCR 113)
Source: PLoS One. 2025 Apr 24;20(4):e0321747. doi: 10.1371/journal.pone.0321747 (PMC12021170; doi:10.1371/journal.pone.0321747)
Supplement: S1 File — (PDF) [file pone.0321747.s001.pdf]

Information pré-opératoire avant néphrectomie partielle par modèle 3D personnalisé : évaluation randomisée contrôlée des effets sur l'anxiété des patients (UroCCR 113). - Rein-3D ANXIETY

Code promoteur : CHUBX 2022/18

PROTOCOLE DE RECHERCHE INTERVENTIONNELLE A RISQUES ET CONTRAINTES MINIMES (RIPH2)

Version n°1.0 du 11/04/2023
Numéro ID-RCB : 2023-A00146-39

Cette recherche a obtenu le financement de l'ANR dans le cadre du RHU Digital Urology 3D

Promoteur :
Centre Hospitalier Universitaire de Bordeaux
12, rue Dubernat
33 400 Talence
FRANCE

Investigateur coordonnateur :
Pr Jean-Christophe BERNHARD
CHU de Bordeaux – Hôpital Pellegrin
Service de Chirurgie Urologique et Transplantation
Place Amélie Raba Léon
33076 Bordeaux Cedex

Centre de Méthodologie et de Gestion des données :
Pr. Laura RICHERT
Unité de Soutien Méthodologique à la Recherche Clinique et Epidémiologique du CHU de Bordeaux
Case 75, 146 rue Léo Saignat
33076 Bordeaux cedex
Tel.: 05 57 57 11 81 - Fax: 05 57 57 15 78
Email: laura.richert@chu-bordeaux.fr

Laboratoire de recherche de sciences humaines et sociales
CeDS EA 74-40
Mme Marthe-Aline Jutand
3 ter Place de la Victoire
33076 Bordeaux Cedex
Tel: +33 (0)5 57 57 19 92 / 06 13 05 78 61
Courriel : marthe-aline.jutand@u-bordeaux.fr

Ce protocole a été conçu et rédigé à partir de la version 3.0 du 01/02/2017 du protocole-type du GIRCI SOHO

HISTORIQUE DES MISES A JOUR DU PROTOCOLE

VERSION	DATE	RAISON DE LA MISE A JOUR
1.0	11/04/2023	Version initiale soumise au CPP

PAGE DE SIGNATURE DU PROTOCOLE

« Information pré-opératoire avant néphrectomie partielle par modèle 3D personnalisé : évaluation randomisée contrôlée des effets sur l'anxiété des patients (UroCCR 113) »

Rein-3D ANXIETY

Code promoteur : CHUBX 2022/18

Promoteur
Centre Hospitalier Universitaire de Bordeaux
12, rue Dubernat
33400 Talence

à Talence, le :

Le Directeur Général du CHU de Bordeaux
Y.BUBIEN
Et par délégation, le Directeur de la Recherche Clinique et de l'Innovation,
G.DULUC

à Bordeaux, le :

Investigateur coordonnateur
Pr Jean-Christophe BERNHARD
CHU de Bordeaux – Hôpital Pellegrin
Service de Chirurgie Urologique et Transplantation
Place Amélie Raba Léon
33076 Bordeaux Cedex
Tel : 05 57 82 03 50
Courriel : jean-christophe.bernhard@chu-bordeaux.fr

PRINCIPAUX CORRESPONDANTS

Investigateur coordonnateur
Pr Jean-Christophe BERNHARD
CHU de Bordeaux - Hôpital Pellegrin
Place Amélie Raba Léon
33076 Bordeaux Cedex
Tél. : 05.57.82.06.87 - Fax : 05.56.79.56.51
Courriel : jean-christophe.bernhard@chu-bordeaux.fr

Programme Manager
Mme Solène RICARD
Service d'Urologie et Transplantation Rénale
CHU de Bordeaux – Pellegrin
Tél. : 05 57 82 12 94
Courriel : solene.ricard@chu-bordeaux.fr

Attachée de Recherche Clinique
Mme Clémence MORICE
Service d'Urologie et Transplantation Rénale
CHU de Bordeaux – Pellegrin
Tél. : 05 57 82 23 94- Fax : 05.56.79.56.51
Courriel : clemence.morice@chu-bordeaux.fr

Unité de Soutien Méthodologique à la Recherche Clinique et épidémiologique
Service d'information médicale, Pôle Santé publique, CHU Bordeaux
146 rue Léo Saignat, case n°75
33076 Bordeaux Cedex FRANCE
Tel : 05 57 57 11 29 / 14 42 - Fax : 05 57 57 15 78
Coordination Méthodologique :
Pr Laura RICHERT
Courriel : laura.richert@chu-bordeaux.fr
Biostatisticienne :
Mme Roxane COUERON
Courriel : roxane.coueron@chu-bordeaux.fr

Unité de la Recherche et de l'Innovation en Soins et en Sciences Humaines (URISH)
Mme Hélène HOARAU
Direction des soins et Direction de la Recherche Clinique et de l'Innovation
12, rue Dubernat
33404 Talence Cedex
Tél. : 06 37 83 92 85
Courriel : helene.hoarau@chu-bordeaux.fr

Promoteur
Centre Hospitalier Universitaire de Bordeaux
12 rue Dubernat
33 400 Talence
FRANCE

Responsable de la recherche au niveau du promoteur
M. Gilles DULUC - Directeur de la Recherche Clinique et de l'Innovation
Dr Anne GIMBERT - Responsable « Promotion interne »
Tél : 05 57 82 08 34 – Fax : 05 56 79 49 26
Courriel : anne.gimbert@chu-bordeaux.fr

Responsable d'Etudes Cliniques
Mme Aline DOUBLET
Direction de la Recherche Clinique et de l'Innovation du CHU de Bordeaux
12 rue Dubernat 33404 Talence Cedex
Tél. : 05 57 82 08 53- Fax : 05 56 79 49 26
Courriel : aline.doublet@chu-bordeaux.fr

Unité de sécurité et de vigilance de la recherche clinique
Direction de la recherche clinique et de l'innovation
12, rue Dubernat
33404 Talence Cedex
Tel: 05 57 82 16 26 - Fax: 05 57 82 12 62
vigilance.essais-cliniques@chu-bordeaux.fr

Lab. Culture et Diffusion des Savoirs (CeDS) EA 7440
Mme Marthe-Aline Jutand
Mme Sarah Masanet
Mme Hélène Hoarau
3 ter Place de la Victoire
33076 Bordeaux Cedex
Tel: +33 (0)5 57 57 19 92 / 06 13 05 78 61
Courriel : marthe-aline.jutand@u-bordeaux.fr

Coordinatrice outil UroCONNECT
Mme Anne CALLEDE
Service d'Urologie et Transplantation Rénale
CHU de Bordeaux – Pellegrin
Tél. : 05 57 82 23 94 - Fax : 05.56.79.56.51
Courriel : anne.callede@chu-bordeaux.

SOMMAIRE

1. RESUME DE LA RECHERCHE 7

ABSTRACT 9

2. JUSTIFICATION SCIENTIFIQUE ET DESCRIPTION GENERALE 11

2.1. ETAT ACTUEL DES CONNAISSANCES 11
2.2. HYPOTHESES DE LA RECHERCHE 11
2.3. JUSTIFICATION DES CHOIX METHODOLOGIQUES 11
2.4. RAPPORT BENEFICE / RISQUE 12
2.5. RETOMBEES ATTENDUES 12
2.6. JUSTIFICATION DU FAIBLE NIVEAU D'INTERVENTION 12

3. OBJECTIFS DE LA RECHERCHE 13

3.1. OBJECTIF PRINCIPAL 13
3.2. OBJECTIFS SECONDAIRES 13

4. CONCEPTION DE LA RECHERCHE 13

4.1. SCHEMA DE LA RECHERCHE 13
4.2. METHODES POUR LA RANDOMISATION 14

5. CRITERES D'ÉLIGIBILITE 14

5.1. CRITERES D'INCLUSION 14
5.2. CRITERES DE NON INCLUSION 14
5.3. FAISABILITE ET MODALITES DE RECRUTEMENT 14

6. PROCEDURES DE LA RECHERCHE 15

6.1. PROCEDURE EXPERIMENTALE 15
6.2. PROCEDURE DE COMPARAISON 16

7. CRITERES DE JUGEMENT 16

7.1. CRITERE DE JUGEMENT PRINCIPAL 16
7.2. CRITERES DE JUGEMENT SECONDAIRES 16

8. DEROULEMENT DE LA RECHERCHE 17

8.1. CALENDRIER DE LA RECHERCHE 17
8.2. TABLEAU RECAPITULATIF DU SUIVI PARTICIPANT 18
8.3. VISITE D'INCLUSION 19
8.4. DEMARCHE DE RANDOMISATION 19
8.5. VISITES DE SUIVI 19
8.6. VISITE DE FIN DE LA RECHERCHE 20
8.7. ABANDON ET RETRAIT DE CONSENTEMENT 20
8.8. REGLES D'ARRET DE LA RECHERCHE 21
8.9. DEVIATIONS AU PROTOCOLE 21
8.10. RISQUES ET CONTRAINTES MINIMES LIEES A LA RECHERCHE ET INDEMNISATION EVENTUELLE DES PARTICIPANTS 21

9. GESTION DES ÉVÉNEMENTS INDÉSIRABLES / EFFETS INDESIRABLES /INCIDENTS 21

10. ASPECTS STATISTIQUES 22

10.1. CALCUL DE LA TAILLE D'ETUDE 22
10.2. METHODES STATISTIQUES EMPLOYEES 22
10.1.3 PATIENTS INCLUS DANS L'ANALYSE 23
10.1.5 METHODES STATISTIQUES DESCRIPTIVES 23
10.1.6 METHODES STATISTIQUES COMPARATIVES 23
10.1.7 LOGICIELS UTILISES 23
10.2 PLAN D'ANALYSE 23
10.3.1 DESCRIPTION DES INCLUSIONS, DES DEVIATIONS ET DU SUIVI 23

10.3.2 CARACTERISTIQUES DES PATIENTS A L'INCLUSION 24
10.3.3 ANALYSE DE L'OBJECTIF PRINCIPAL 24
10.3.4 ANALYSE DES OBJECTIFS SECONDAIRES 24

11. SURVEILLANCE DE LA RECHERCHE 25

11.1. Conseil scientifique 25
11.1.1 COMPOSITION 25
11.1.2 RYTHME DES REUNIONS 25
11.1.3 ROLE 25
11.2. Comité indépendant de Surveillance 26

12. DROITS D'ACCES AUX DONNEES ET DOCUMENTS SOURCE 26

12.1. ACCES AUX DONNEES 26
12.2. DONNEES SOURCES 26
12.3. CONFIDENTIALITE DES DONNEES 26

13. CONTROLE ET ASSURANCE QUALITE 26

13.1. CONSIGNES POUR LE RECUEIL DES DONNEES 26
13.2. CONTROLE QUALITE 26
13.3. GESTION DES DONNEES 27
13.3.1 LOGICIEL DE GESTION DE DONNEES 27
13.3.2 SAISIE DES DONNEES 27
13.3.3 CODAGE DES DONNEES 27
13.3.4 CONTROLES DES DONNEES 28
13.3.5 TRANSFERT DES DONNEES 28
13.4. AUDIT ET INSPECTION 28

14. CONSIDERATIONS ETHIQUES ET REGLEMENTAIRES 28

15. CONSERVATION DES DOCUMENTS ET DES DONNEES RELATIVES A LA RECHERCHE 29

16. RAPPORT FINAL 29

17. REGLES RELATIVES A LA PUBLICATION 30

17.1. COMMUNICATIONS SCIENTIFIQUES 30
17.2. COMMUNICATION DES RESULTATS AUX PARTICIPANTS 30
17.3. CESSION DES DONNEES 30

REFERENCES BIBLIOGRAPHIQUES 31

ANNEXES 32

ANNEXE 1 : FICHE INFO-PATIENT DE L'AFU 33
ANNEXE 2 : ECHELLE STAI ETAT 42
ANNEXE 3 : ECHELLE STAI TRAIT 43
ANNEXE 4 : ECHELLE LITERATIE HLSEU 16 44
ANNEXE 5 : ECHELLE QUALITE DE VIE – EQ-5D-5L 46
ANNEXE 6 : QUESTIONNAIRE DE WAKE 48
ANNEXE 7 : CONSENTEMENT UROCCR 49

LISTE DES ABREVIATIONS

AFU : Association Française d'Urologie

CeDS : Culture et Diffusion Des Savoirs

CMG : Centre de Méthodologie et de Gestion des données

CNIL : Commission Nationale de l'Informatique et des Libertés

CPP : Comité de Protection des Personnes

CREDIM : Centre de Recherche et Développement en Informatique Médicale

DRCI : Direction de la Recherche Clinique et de l'Innovation

HLSEU-Q16 : European Health Literacy Survey Questionnaire

RAAC : Réhabilitation Accélérée en Chirurgie

RAPN : Robotic Assisted Partial Nephrectomy

STAI : State-Trait Anxiety Inventory

URISH : Unité de la Recherche et de l'Innovation en Soins et Sciences Humaines

UroCCR : Réseau français de recherche sur le cancer du rein

USMR : Unité de Soutien Méthodologique à la Recherche Clinique et Epidémiologique

1. RESUME DE LA RECHERCHE

**PROMOTEUR**	CHU de Bordeaux
**INVESTIGATEUR COORDONNATEUR**	Pr Jean-Christophe BERNHARD
**ACRONYME ET TITRE**	R3DP-A – Information pré-opératoire avant néphrectomie partielle par modèle 3D personnalisé : évaluation randomisée contrôlée des effets sur l'anxiété des patients (UroCCR 113).
**TITRE SIMPLIFIE**	Rein 3D – Anxiety
**JUSTIFICATION / CONTEXTE**	L'annonce diagnostique d'une pathologie tumorale et la perspective d'une intervention chirurgicale peuvent générer angoisses et retentissement psychologique majeur.<br>La recherche d'outils permettant de limiter l'anxiété à l'approche de l'intervention chirurgicale est très peu développée à l'heure actuelle. Cependant elle paraît être un enjeu crucial dans la prise en charge globale de ces malades afin d'améliorer le vécu de l'hospitalisation et de l'intervention et diminuer les prescriptions médicamenteuses anxiolytiques. La maîtrise de l'anxiété pré-opératoire participe aussi à faciliter la mise en œuvre des preceptes de réhabilitation améliorée en chirurgie (RAAC) en diminuant la consommation de médicaments psychotropes et d'antalgiques
**OBJECTIFS**	**Objectif principal** : Evaluer l'effet de l'utilisation d'un modèle tridimensionnel personnalisé du rein du patient lors d'une visite pré-opératoire d'information, sur l'anxiété pré-opératoire, en comparant un groupe avec son propore modèle virtuel et un groupe son propore avec modèle imprimé à un groupe sans modèle (groupe contrôle).<br><br>**Objectifs secondaires** :<br>1. Evaluer l'évolution du niveau d'anxiété dans chacun des trois groupes entre l'inclusion et J15 post-opératoire<br>2. Evaluer l'évolution du niveau de litteratie en santé dans chacun des trois groupes entre l'inclusion et J15 post-opératoire<br>3. Evaluer le sentiment de compréhension de la maladie et du choix du traitement en pré-opératoire dans chacun des trois groupes<br>4. Evaluer l'évolution de la qualité de vie dans chacun des trois groupes<br>5. Décrire la durée moyenne de la visite d'information pré-opératoire dans chacun des trois groupes
**SCHEMA DE LA RECHERCHE**	Essai clinique multicentrique contrôlé, de supériorité, randomisé avec un ratio 1 :1 : 1 en 3 bras parallèles, sans insu
**CRITERES D'INCLUSION**	- Patients adultes (≥ 18 ans)<br>- Prise en charge chirurgicale programmée par néphrectomie partielle laparoscopique avec assistance robotique pour tumeur du rein<br>- Tumeur du rein unilatérale ou 1ère chirurgie d'une atteinte bilatérale<br>- Consentement exprimé pour intégration de la cohorte UroCCR,<br>- Consentement exprimé pour participation à l'étude 3D Anxiety.<br>- Patients affiliés ou bénéficiaires d'un régime de sécurité sociale

**CRITERES DE NON INCLUSION**	- Refus de consentement ou de participation<br>- Difficultés à comprendre et à s'exprimer en français<br>- Patient sous tutelle ou curatelle<br>- Absence de scanner préopératoire disponible pour la modélisation 3D
**STRATEGIES DE LA RECHERCHE**	Lors de l'inclusion dans l'étude les participants seront randomisés en 3 bras, correspondants aux différents supports d'information utilisés lors d'un temps d'échange programmé avec le patient. :<br>1. Groupe modèle 3D Virtuel : temps d'échange avec pour support d'information le modèle tridimensionnel virtuel du rein à opérer<br>2. Groupe modèle 3D Imprimé : temps d'échange avec pour support d'information le modèle tridimensionnel physique imprimé du rein à opérer<br>*3.* Groupe contrôle : temps d'échange avec pour support d'information la fiche Info-patient de l'Association Française d'Urologie (AFU)
**CRITERES DE JUGEMENT**	**Critère de jugement principal :** Score d'anxiété moyen pré-opératoire par bras, mesuré à J-1 de la chirurgie par autoquestionnaire STAI-état<br><br>**Critères de jugement secondaires :**<br>1. Evolution du score d'anxiété moyen par bras, mesuré par auto-questionnaire STAI-état aux trois temps de suivi dans l'étude et ajustée sur le score STAI-Trait à l'inclusion<br>2. Evolution du score moyen du questionnaire de littératie en santé HLSEU-Q16 entre la mesure à l'inclusion et à 15 jours post-opératoire par bras<br>3. Score moyen pré-opératoire de compréhension de la maladie et du choix du traitement (Wake, 2019 ; mesuré à J-1 de la chirurgie) par bras<br>4. Evolution du score moyen de EQ-5D-5L entre l'inclusion et J15 post-opératoires par bras<br>5. Durée moyenne du temps d'échange pré-opératoire dans chacun des trois groupes
**TAILLE D'ETUDE**	234 patient (78 dans chaque bras)
**DUREE DE LA RECHERCHE**	Durée de la période d'inclusion : 24 mois<br>Durée de participation de chaque participant : 2 à 4 mois<br>Durée totale de la recherche : 26 à 28 mois
**ANALYSE STATISTIQUE DES DONNEES**	L'analyse principale sera effectuée en intention-de-traiter (ITT).<br>Le STAI-Etat pré-opératoire sera décrit globalement et par groupes de procédure, en utilisant les méthodes statistiques descriptives standard (effectif, moyenne, écart-type, médiane, étendue et étendue interquartile).<br>L'analyse principale comparative sera effectuée au risque d'erreur global $\alpha = 5\%$ soit un seuil de p-valeur à 2.5% (correction de Bonferroni) pour chacune des deux comparaisons (« groupe Modèle 3D Virtuel vs Groupe contrôle » et « groupe Modèle 3D imprimé vs Groupe contrôle »), sans ajustement (test de Student ou Wilcoxon). Nous réaliserons ensuite un modèle de régression linéaire à effets mixtes pour estimer les différences entre les bras de randomisation sur le score STAI-Etat pré-opératoire en ajustant sur le centre (facteur de stratification de la randomisation), le score STAI-Etat à l'inclusion, le score STAI-Trait à l'inclusion et en incluant les termes d'interactions adéquats.<br>Une analyse secondaire sera réalisée sur la population per-protocole.
**RETOMBEES ATTENDUES**	Mise en évidence d'un bénéfice apporté par les modèles tridimensionnels rénaux sur l'état d'anxiété pré-opératoire, la qualité de vie et la littératie, justifiant l'utilisation de ces modèles dans le parcours de soin des patients traités par néphrectomie partielle. Amélioration du vécu de l'hospitalisation et de l'intervention.

ABSTRACT

This research has been registered in http://www.clinicaltrials.gov/

Effect on pre-operative anxiety of a personalized three-dimensional kidney model prior to nephron-sparing surgery for renal tumor. R3DP-A

The University Hospital of Bordeaux is the sponsor of this research.
This research will be conducted with the support of ANR-21-RHUS-0015.

- **Brief summary :** We aimed to show the impact of showing and handling a three-dimensional modelization of a patient's tumoral kidney the day before his nephron-sparing surgery. The main outcome measure was the effect on anxiety assessed via the STAI state score. 3 types of pre-operative information were compared (3D virtual model, 3D printed model, and information) using a randomization.
- **Detailed description:** Announcement of serious illnesses and especially cancerous disease has major impacts on one's psychological field. Need of surgery can create even other fears due to its unknown nature. Use of tools to decrease anxiety and enhance understanding prior to surgery is a key point in comprehensive care that is way not enough promoted for now. In the field of surgery, available methods to deal with pre-operative anxiety are massively represented by anxiolytics drugs, only few other devices were evaluated.

  Technological development in healthcare has seen the rising of three-dimensional media, especially in surgery, for procedure planning. There is no doubt that this tool is effective in helping surgeons regarding the studies published within the past few years but there is no such evidence concerning the benefit for patients. Some authors report improvement of patients understanding and great satisfaction referring to the usefulness of this tool. But none of these papers evaluated anxiety.

  The goal of our research is to measure the effect of a personalized three-dimensional model of patients' tumoral kidney on peri-operative anxiety.

  For this purpose we will randomly assign a total of 234 (78*3) patients planned to have a nephron-sparing surgery for kidney tumor in 3 different groups. Inside the 2 interventional groups, patients' kidneys will be modelled in three dimensions, but in one of the two groups, the model will be 3D-printed. The control group will include patients for whom the pre-operative information will be made based on standardized information letter from the French Urological Asssociation.

  Before surgery, during a preoperative therapeutic education consultation, 3D models will be presented to the patients according to the allocated study group. All patients will have to complete questionnaires to assess their anxiety level, their quality of life, their health literacy level and their understanding of disease and surgical procedure.

  One month after surgery, during the post-operative consultation, patients will also fill out the different questionnaires to assess their anxiety level, quality of life, and level of litteracy.

- **Primary outcome:** To evaluate the effect of using a personalized three-dimensional model of the patient's kidney during a pre-operative information visit on pre-operative anxiety by comparing a group with its own virtual model and a group with its own printed model to a group without a model (control group).
- **Secondary outcomes:**

1. To evaluate the evolution of the anxiety level in each of the three groups between inclusion and D15 post-op
2. To evaluate the evolution of the level of health literacy in each of the three groups between inclusion and D15 post-op
3. To assess the feeling of understanding of the disease and the choice of treatment preoperatively in each of the three groups
4. To evaluate the evolution of quality of life in each of the three groups
5. Describe the average duration of the pre-operative information visit in each of the three groups

- **Study design:** Multicenter, superiority, randomized, controlled clinical trial with a 1:1:1 ratio in 3 parallel arms, without blinding
- **Eligibility criteria:**

- Adult patients (≥ 18 years of age)

- Scheduled surgical management by laparoscopic partial nephrectomy with robotic assistance for kidney tumor

- Unilateral kidney tumor or 1st surgery for bilateral involvement

- Consent expressed for integration of the UroCCR cohort,

- Expressed consent for participation in the 3D Anxiety study.

- Patients affiliated or benefiting from a social security plan

- **Arm number or label and arm type**:
    - Group 1 : Virtual 3D model group: exchange time with the virtual 3D model of the kidney to be operated on as information support
    - Group 2 : 3D Printed Model Group: exchange time with the printed three-dimensional model of the kidney to be operated on as information support
    - Group 3 : Control group: discussion time with the patient information sheet of the French Association of Urology (AFU) as information support
- **Number of subjects:** 234 subjects

Statistical analyses: The main analysis will be performed in intention-to-treat (ITT).

The preoperative STAI-State will be described globally and by procedure groups, using standard descriptive statistical methods (number of patients, mean, standard deviation, median, range and interquartile range).

The main comparative analysis will be performed at the overall risk of error $\alpha = 5\%$, i.e., a p-value threshold of 2.5% (Bonferroni correction) for each of the two comparisons ("Virtual 3D Model group vs. Control group" and "Printed 3D Model group vs. Control group"), without adjustment (Student's or Wilcoxon test). We will then perform a linear mixed-effects regression model to estimate the differences between the randomization arms on the preoperative STAI-Status score by adjusting for center (stratification factor of the randomization), STAI-Status score at inclusion, STAI-Treatment score at inclusion, and by including appropriate interaction terms.

A secondary analysis will be performed on the per-protocol population.

- **Key-words:** Kidney Neoplasms, Renal-Cell Carcinoma, Nephron-Sparing Surgery, 3D Modeling, 3D Printing, Prospective Studies, Random Allocation, Anxiety, STAI, Personalized Medicine

2. JUSTIFICATION SCIENTIFIQUE ET DESCRIPTION GENERALE

2.1. ETAT ACTUEL DES CONNAISSANCES

2.1.1. SUR LA PATHOLOGIE

L'annonce diagnostique d'une pathologie tumorale et la perspective d'une intervention chirurgicale peuvent générer angoisses et retentissement psychologique majeurs (Rusznniewski & Rabier, 2015).

La recherche d'outils permettant de limiter l'anxiété à l'approche de l'intervention chirurgicale est très peu développée à l'heure actuelle. Cependant elle paraît être un enjeu crucial dans la prise en charge globale de ces malades afin d'améliorer le vécu de l'hospitalisation et de l'intervention et diminuer les prescriptions médicamenteuses anxiolytiques. La maîtrise de l'anxiété pré-opératoire participe aussi à faciliter la mise en œuvre des preceptes de réhabilitation améliorée en chirurgie (RAAC) en diminuant la consommation de médicaments psychotropes et d'antalgiques.

2.1.2. SUR LES TRAITEMENTS/STRATEGIES/PROCEDURES DE REFERENCE ET A L'ETUDE

En 2015, une étude pilote a testé l'utilisation d'un modèle 3D de rein personnalisé, auprès de patients atteints d'un cancer du rein non métastatique lors d'une consultation chirurgicale (Bernhard et al., 2016). Il en ressort que ce type de modèle 3D pourrait être employé comme outil de médiation éducative, facilitant ainsi la compréhension de la pathologie et des enjeux chirurgicaux, tout en améliorant la satisfaction des patients.

2.2. HYPOTHESES DE LA RECHERCHE

- **Hypothèse principale :**

L'hypothèse de cette étude est que l'utilisation de la représentation tridimensionnelle (modèle virtuel ou modèle imprimé palpable) du rein du patient atteint de tumeur rénale, candidat à un traitement conservateur, permet de réduire l'anxiété pré-opératoire, par comparaison à d'autres outils d'information tels que la fiche Info-Patient de l'AFU (standard de soin).

- **Hypothèses secondaires :**

L'utilisation de la représentation personnalisée, en 3D, du rein tumoral permet d'améliorer la compréhension de la maladie, le niveau global de litteratie en santé et la qualité de vie du patient.

2.3. JUSTIFICATION DES CHOIX METHODOLOGIQUES

Un essai randomisé comparatif, multicentrique, de supériorité sera mené. Les participants seront randomisés avec un ratio 1 :1 :1 en trois groupes parallèles :

- Groupe 1 : Groupe Modèle 3D Virtuel : utilisation du modèle 3D Virtuel lors du temps d'échange pré-opératoire
- Groupe 2 : Groupe Modèle 3D imprimé : utilisation du modèle 3D imprimé lors du temps d'échange pré-opératoire
- Groupe 3 : Groupe contrôle : utilisation de la fiche Info-Patient de l'AFU (Annexe 1) lors du temps d'échange pré-opératoire, pas d'utilisation d'un modèle 3D.

Le critère de jugement principal est le score d'anxiété moyen pré-opératoire par bras, défini à l'aide de l'autoquestionnaire STAI-état (Annexe 2). Le remplissage de cet autoquestionnaire se fera à J-1 avant la chirurgie.

Le STAI-Etat est une des deux échelles du STAI Y, échelles destinées à évaluer l'anxiété momentanée et l'anxiété habituelle. La version française validée du « State-Trait Anxiety Inventory Form Y » (version anglaise : Spielberger, Gorsuch, Lushene, Vagg, & Jacobs, 1983 ; version française Spielberger & Bruchon-Schweitzer, 1993) sera utilisée. Chacune des deux échelles est composée de 20 items. **L'échelle d'anxiété STAI-Etat** permet d'évaluer les sentiments d'inquiétude, d'appréhension, de tension et de nervosité du sujet au moment de la passation. C'est un indicateur des modifications transitoires de l'anxiété provoquées par des situations thérapeutiques ou aversives, ici la situation pré-opératoire. Pour cette échelle, l'individu doit choisir à quel point l'item lui correspond entre « non », « plutôt non », « plutôt oui » et « oui ». Chaque item étant évalué sur une échelle de Likert en 4 points, le score total varie entre 20 et 80. Pour faciliter l'interprétation, les scores peuvent être classés en 5 niveaux : inférieur ou égal à 35 (très faible), de 36

à 45 (faible), de 46 à 55 (moyen), de 56 à 65 (élevé) et supérieur à 65 (très élevé). Le STAI n'est pas un outil diagnostique de l'anxiété, mais un outil d'évaluation.
L'échelle STAI-Etat a été choisie comme critère de jugement principal compte tenu de : i) sa capacité de mesurer un état d'anxiété transitoire, adapté à notre contexte d'anxiété pré-opératoire ; ii) son utilisation antérieure dans la litérature, permettant des poser des hypothèses statistiques pour le calcul d'effectif ; et iii) l'existence d'une version française validée.

Compte tenu de la nature des interventions testées (outils utilisés lors d'une visite d'information) et d'un critère de jugement patient-centré (anxiété recueillie par auto-questionnaire), la mise en place d'un insu dans cet essai n'est pas envisagée.

Tenant en compte le schéma à 3 bras comprenant 2 bras expérimentaux (avec modèles 3D) qui seront chacun comparés au bras contrôle, un ajustement Bonferonni sera réalisé pour tenir compte des 2 tests statistiques pour l'analyse du critère de jugement principal.

2.4. RAPPORT BENEFICE / RISQUE

Les bénéfices attendus sont, en premier lieu, l'amélioration de l'anxiété et de la qualité de vie des patients ainsi que l'amélioration de la compréhension de l'information pré-opératoire, conditions cruciales pour une meilleure préparation à la chirurgie. Celle-ci a pour conséquence une potentielle diminution de la douleur post-opératoire permettant une réhabilitation plus rapide.
L'intervention de l'étude consistant à utiliser un modèle 3D personnalisé du rein permet un échange avec le patient propice à l'évocation d'interrogations, questions en suspens sur la prise en charge ou la maladie. Pendant le suivi, le participants répondra à des auto-questionnaires (non diagnostiques), il n'existe aucun risque physique pour la participation à cette étude. Néanmoins, il est possible que le fait de poser des questions en lien avec l'anxiété ouvre sur d'autres interrogations.
En effet, les questions interrogent le ressenti du patient, on peut imaginer que le patient démarre alors un cheminement de type introspectif qu'il n'avait pas eu auparavant et qui peut avoir des conséquences psychologiques que l'on estime modérées. Il est à souligner que la prise en charge globale du patient s'inscrit dans un réseau de soins comportant un accompagnement soignant rapproché durant l'hospitalisation et des interlocuteurs disponibles tout au long de son suivi avec possibilité d'avoir recours à un psychologue.

2.5. RETOMBEES ATTENDUES

Mise en évidence d'un bénéfice apporté par les modèles tridimensionnels rénaux sur l'état d'anxiété pré-opératoire, la qualité de vie et la littératie, justifiant l'utilisation de ces modèles dans le parcours de soin des patients traités par néphrectomie partielle. Amélioration du vécu de l'hospitalisation et de l'intervention..

2.6. JUSTIFICATION DU FAIBLE NIVEAU D'INTERVENTION

Afin d'établir l'impact de l'utilisation du modèle 3D virtuel ou imprimé sur l'anxiété des patients, au circuit de prise en charge habituel vient s'ajouter une **consultation d'échange et d'information préopératoire**, délivrée par un médecin. Ces consultations seront l'occasion de répondre aux interrogations du patient et d'échanger en conséquence sur les modalités de la chirurgie à venir en s'appuyant en standard sur la fiche Info-Patient de l'AFU (Annexe 1) ou bien sur les modélisations rénales (2 types de modélisation sont proposés).
Les autres procédures spécifiques à l'étude ne concernent que le recueil de nos critères de jugement par le biais d'auto-questionnaires.
Les prises en charge médicale et chirurgicale du patient, réalisées en routine, demeurent inchangées sur le fond.

3. OBJECTIFS DE LA RECHERCHE

3.1. OBJECTIF PRINCIPAL

L'objectif principal de cette étude est d'évaluer l'effet de l'utilisation d'un modèle tridimensionnel personnalisé du rein du patient, lors d'une visite pré-opératoire d'information, sur l'anxiété pré-opératoire, en comparant un groupe avec modèle virtuel et un groupe avec modèle imprimé à un groupe sans modèle (groupe contrôle).

3.2. OBJECTIFS SECONDAIRES

1 Evaluer l'évolution du niveau d'anxiété dans chacun des trois groupes entre l'inclusion et J15 post-opératoire
2 Evaluer l'évolution du niveau de litteratie en santé dans chacun des trois groupes entre l'inclusion et J15 post-opératoire
3 Evaluer le sentiment de compréhension de la maladie et du choix du traitement en pré-opératoire dans chacun des trois groupes
4 Evaluer l'évolution de la qualité de vie dans chacun des trois groupes
5 Décrire la durée moyenne du temps d'échange pré-opératoire dans chacun des trois groupes

4. CONCEPTION DE LA RECHERCHE

4.1. SCHEMA DE LA RECHERCHE

Etude interventionnelle comparative, multicentrique, de supériorité, randomisée en 3 groupes parallèles, sans insu.

Les participants seront randomisés avec un ratio 1 :1 :1 à trois groupes :

- Groupe 1 : Groupe Modèle 3D virtuel ;
- Groupe 2 : Groupe Modèle 3D imprimé ;
- Groupe 3 : Groupe contrôle bénéficiant d'une information à partir de la fiche Info-Patient de l'AFU (Annexe 1).

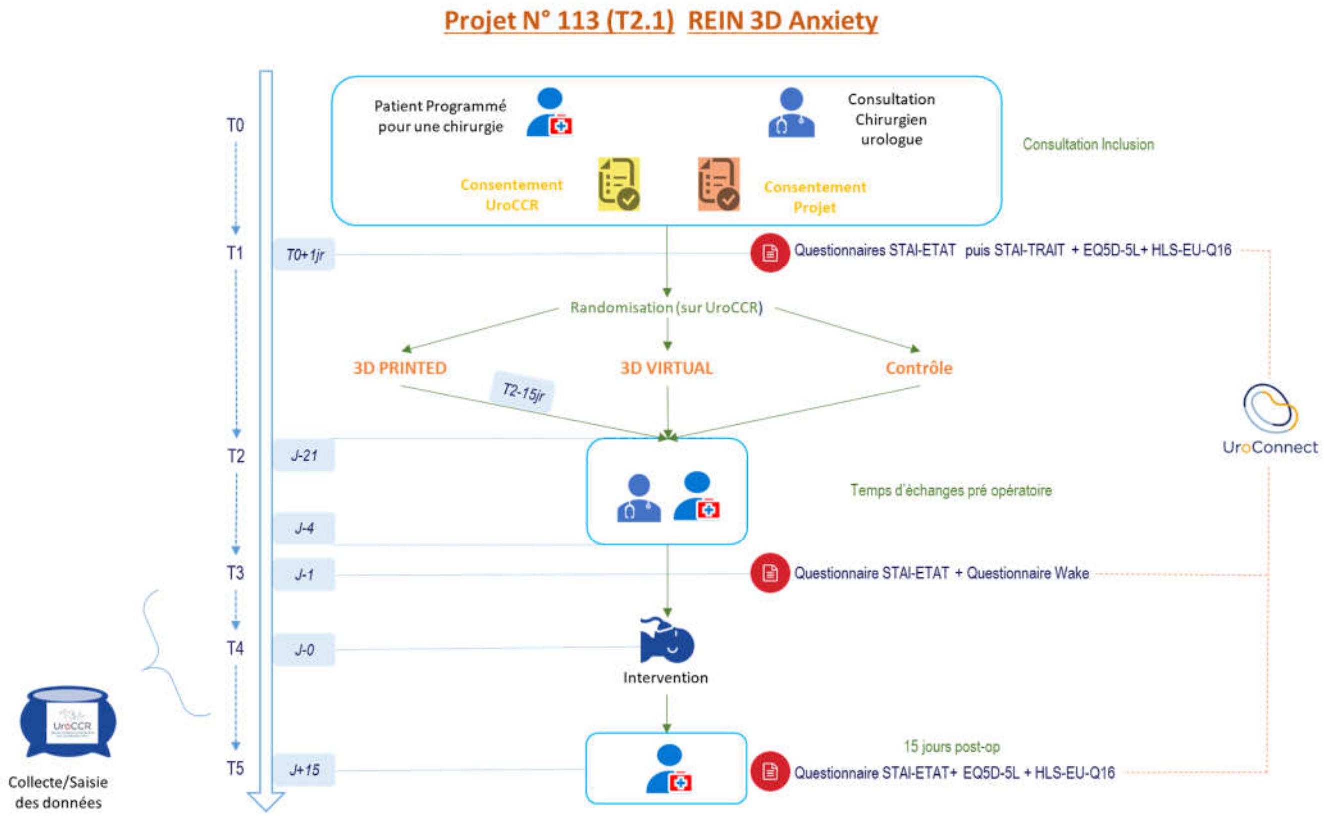

4.2. METHODES POUR LA RANDOMISATION

La liste de randomisation est établie par le statisticien du Centre de Méthodologie et de Gestion des données (USMR du CHU de Bordeaux) avant le début de la recherche.

Les effectifs des 3 groupes de stratégie sont équilibrés avec un ratio 1 :1 :1. La randomisation sera stratifiée sur les centres investigateurs. Un document décrivant la procédure de randomisation est conservé de manière confidentielle au sein du Centre de Méthodologie et de Gestion des données.

La randomisation se fera directement dans l'interface web de la base de données UroCCR, lorsque le patient est inclus.

La procédure à suivre en pratique pour la randomisation d'un patient est décrite dans le paragraphe « 8.4. Démarche de randomisation » du protocole.

5. CRITERES D'ÉLIGIBILITE

5.1. CRITERES D'INCLUSION

- Patients adultes (≥ 18 ans)
- Prise en charge chirurgicale programmée par néphrectomie partielle laparoscopique avec assistance robotique pour tumeur du rein
- Tumeur du rein unilatérale ou 1ère chirurgie d'une atteinte bilatérale
- Consentement exprimé pour intégration de la cohorte UroCCR,
- Consentement exprimé pour participation à l'étude 3D Anxiety.
- Patients affiliés ou bénéficiaires d'un régime de sécurité sociale

5.2. CRITERES DE NON INCLUSION

- Refus de consentement ou de participation
- Difficultés à comprendre et à s'exprimer en français
- Patient sous tutelle ou curatelle
- Absence de scanner préopératoire disponible pour la modélisation 3D

5.3. FAISABILITE ET MODALITES DE RECRUTEMENT

5.3.1. FAISABILITE

Depuis 2011, sous l'égide de l'INCa, un Réseau national de recherche et d'évaluation des pratiques de prise en charge du cancer du Rein a été déployé (www.uroccr.fr – NCT03293563). La diversification des modalités de prise en charge des tumeurs du rein et le déploiement du concept de concertation pluridisciplinaire, a conduit à une adaptation dynamique de nos outils et procédures de recherche à la réalité des soins actuellement portés aux patients atteints de Cancer du rein. Ce Réseau collaboratif multidisciplinaire, s'appuyant sur une Base Clinico-Biologique spécifique (base UroCCR), est déployé sur 44 centres Français (CHU, CH, Centres privés, CLCC).

La base de données prospective UroCCR permet l'acquisition continue et sécurisée de données de *vraie vie* (données démographiques et cliniques, données associées aux échantillons biologiques mais également résultats expérimentaux). Elle représente un atout majeur pour l'évaluation prospective des pratiques de prise en charge en vie réelle par la création d'un véritable parcours intégré associant Soin et Recherche.

Plus de 15 000 patients à ce jour ont été inclus prospectivement, faisant d'UroCCR l'une des plus grosses cohortes actives internationales de patients atteints de cancer du rein.

Actuellement, 29 projets ancillaires sont menés dans le cadre du Réseau UroCCR mettant à profit la structuration du réseau et l'interface informatique à disposition. Il s'agit de projets rétrospectifs sur données existantes, de projets prospectifs interventionnels, ou de recherches translationnelles. Pour tous ces projets de recherche et de soins courants, l'équipe coordinatrice d'UroCCR, le CREDIM (développement informatique) ainsi que tous les utilisateurs d'UroCCR

(plus de 320 actifs) œuvrent ensemble avec pour objectif commun d'évaluer, sécuriser et améliorer les prises en charges de nos patients.

Cette évaluation sera menée sur 6 centres du réseau français de Recherche sur le Cancer du Rein UroCCR.

5.3.2. Modalites de recrutement

Dans les centres participants, seront inclus tous les patients adultes pris en charge dans les services de chirurgie Urologique, répondant aux critères d'inclusion et programmés pour une néphrectomie partielle par voie laparoscopique avec assistance robotique pour tumeur du rein. .

Le recrutement aura lieu au moment de la consultation d'urologie et de la planification d'une néphrectomie partielle pour tumeur. La participation à l'étude sera proposée à tous les participants consécutifs remplissant les critères d'éligiblité.

6. PROCEDURES DE LA RECHERCHE

6.1. PROCEDURE EXPERIMENTALE

6.1.1. Groupe Modele 3D Virtuel

La modélisation tridimensionnelle du rein atteint à partir du scanner est réalisée à l'aide du logiciel Synapse 3D (Fujifilm) par l'équipe chirurgicale.

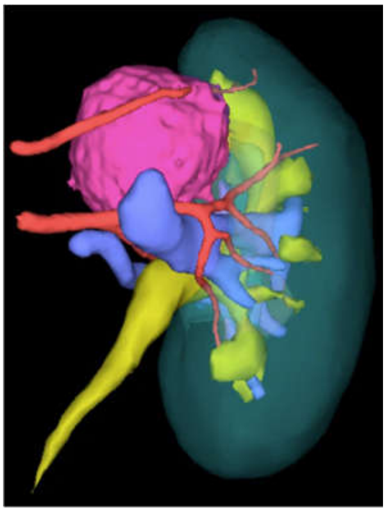

Exemple de modélisation tridimensionnelle virtuelle

Avant l'intervention, un temps d'échange entre chirurgien et patient est organisé entre J-21 et J-4 avant l'opération. Ce temps permettra de répondre aux interrogations du patient en lien avec sa prise en charge et l'intervention à venir en utilisant le modèle trimidensionnel virtuel comme support didactique.

6.1.2. Groupe Modele 3D Imprime

L'étape de modélisation tridimensionnelle est identique à celle réalisée pour le Groupe Modèle 3D Virtuel.

L'impression de ce modèle est ensuite réalisée à l'aide de l'imprimante J750 Stratasys acquise en partenariat avec l'IUT de Bordeaux et située sur le site de l'IUT(Envoi des fichiers 3D (.stl) anonymisés à l'ingénieur de l'IUT en charge des impressions). Le délai d'obtention du modèle 3D imprimé est d'environ 15 jours après la réalisation du scanner.

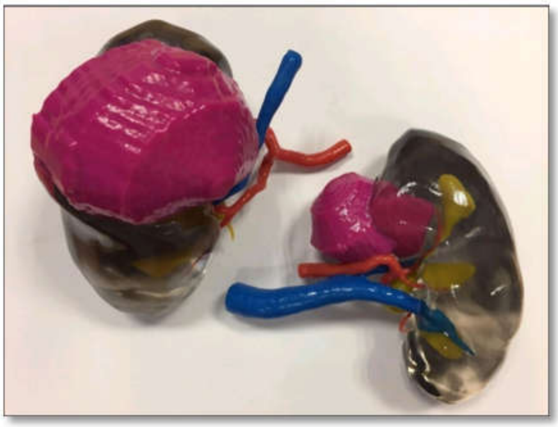

Exemple de modélisation tridimensionnelle imprimée

Avant l'intervention, un temps d'échange entre chirurgien et patient est organisé entre J-21 et J-4 avant l'opération. Ce temps permettra de répondre aux interrogations du patient en lien avec sa prise en charge et l'intervention à venir en utilisant le modèle trimidensionnel imprimé comme support didactique. Le patient peut alors prendre en main et manipuler ce modèle.

6.2. PROCEDURE DE COMPARAISON

Le groupe témoin (Groupe 3) ne dispose d'aucun modèle rénal personnalisé mais bénéficie d'un temps d'échange entre chirurgien et patient entre J-21 et J-4. Ce temps permettra de répondre aux interrogations du patient en lien avec sa prise en charge et l'intervention à venir en utilisant la Fiche-Info de l'AFU comme support didactique.

7. CRITERES DE JUGEMENT

7.1. CRITERE DE JUGEMENT PRINCIPAL

Le critère de jugement principal est le score d'anxiété moyen pré-opératoire par bras, mesuré à J-1 de la chirurgie par autoquestionnaire STAI-Etat (Annexe 2).

Le STAI-Etat sera recueilli avant l'opération (J-1) pour chaque participant, via l'application Uroconnect ou un support papier pour les patients ne disposant pas de smartphone ou d'ordinateur. Les patients recevront une notification sur leur mobile ou un appel téléphonique de l'attaché de recherche clinique pour leur rappeler de compléter le questionnaire.
Le temps de passation de l'échelle est estimé entre 5 et 10 minutes.

7.2. CRITERES DE JUGEMENT SECONDAIRES

Les critères de jugements secondaires sont définis pour répondre aux différents objectifs secondaires :

1. **Evolution du score d'anxiété moyen par bras, mesuré par auto-questionnaire STAI-Etat aux trois temps de suivi dans l'étude et ajustée sur le score STAI-Trait à l'inclusion**

   Pour évaluer l'évolution du score d'anxiété dans chacun des trois groupes, l'échelle STAI-Etat sera également recueillie (avant la mesure de l'echelle STAI-Trait) à l'inclusion et à J15 post-opération (en plus du temps pré-opératoire du critère de jugement principal).

   A l'inclusion l'échelle STAI-Trait sera complétée(après l'échelle STAI-Etat). Elle permet d'évaluer les sentiments d'appréhension, la tension, la nervosité et l'inquiétude que le sujet ressent habituellement (annexe 3). Cette échelle a pour but de repérer l'anxiété comme disposition stable. Pour cette échelle, le participant doit répondre en indiquant si chaque item est vrai « presque jamais », « parfois », « souvent » ou « presque toujours ». Les individus présentant un score élevé à l'échelle d'Anxiété STAI-Trait seront plus sujet à

manifester un score plus élevé à l'échelle d'Anxiété STAI-Etat du fait d'une vision plus anxieuse de leur environnement (Spielberger & Bruchon-Schweitzer, 1993).
Le temps de passation de l'échelle est estimé entre 5 et 10 minutes, soit un temps maximum de 20 minutes pour la passation du STAI complet à l'inclusion.

2. **Evolution du score moyen du questionnaire de littératie en santé HLSEU-Q16 entre la mesure à l'inclusion et à 15 jours post-opératoire par bras**

Le questionnaire **HLSEU-Q16** sera utilisé à l'inclusion et à J15 post-opératoire. Ce questionnaire est une version courte du « European Health Literacy Survey Questionnaire » (Sørensen et al., 2013) basé sur un modèle incluant 4 compétences liées au traitement de l'information en santé : l'accès, la compréhension, l'évaluation et l'application de l'information sur la santé (annexe 4).
Ces quatre compétences sont explorées dans trois contextes de santé : soins en santé, préventions des maladies et promotion de la santé. Le HLSEU-Q16 est composé de 16 items. Toutes les dimensions sont explorées à l'exception de la dimension « appliquée l'information en promotion de la santé ». Cette version courte a été validée en français par Rouquette en 2018 (Rouquette et al., 2018).
Chaque item est noté sur une échelle à quatre modalités : « très facile », « facile », « difficile » et « très difficile ». Pour la cotation, le score à chaque item est ramené à un score binaire, les modalités « très facile » et « facile » cotant pour 1 alors que « difficile » et « très difficile » pour 0.
Le score total peut alors varier de 0 à 16. Pour faciliter l'interprétation, ils peuvent être classés en trois niveaux de littératie : inférieur ou égal à 8 traduisant un niveau inadéquat, de 9 à 12 traduisant un niveau problématique et strictement supérieur à 12 un niveau adéquat.

3. **Score moyen pré-opératoire de compréhension de la maladie et du traitement choisi (Wake, 2019 ; mesuré à J-1 de la chirurgie) par bras**

Pour évaluer le **sentiment de compréhension de la maladie et du choix du traitement** dans chacun des trois groupes, le **questionnaire de Wake** (Wake, 2019) sera utilisé dans sa traduction française réalisée dans le cadre de ce protocole (annexe 6).

4. **Evolution du score moyen de EQ-5D-5L entre l'inclusion et J15 post-opératoires par bras**

Le questionnaire EQ-5D-5L (Annexe 5) permet de mesurer la qualité de vie des participants (Herdman et al., 2011). Pour ce faire, cinq dimensions : la mobilité, l'autonomie de la personne, l'impact sur les activités courantes, la douleur et la gêne et l'anxiété et la dépression, sont étudiées. Pour chacune d'entre-elles le sujet doit indiquer laquelle des cinq propositions lui correspond le mieux (allant de « je n'ai pas de problème » à « je suis incapable de »). En complément de cette mesure, le sujet doit évaluer son état de santé sur une échelle visuelle allant de 0 (la pire santé) à 100 (la meilleure santé). Traduite dans plus de 200 langues différentes y compris en français (Andrade et al., 2020), ce questionnaire est l'un des plus utilisés dans le monde.

5. **Durée moyenne du temps d'échange pré-opératoire dans chacun des trois groupes**

8. DEROULEMENT DE LA RECHERCHE

8.1. CALENDRIER DE LA RECHERCHE

- Durée de la période d'inclusion : 24 mois
- Durée de participation de chaque participant : de 2 à 4 mois
- Durée totale de la recherche : 26 à 28 mois

8.2. TABLEAU RECAPITULATIF DU SUIVI PARTICIPANT

- **Tableau récapitulatif du suivi participant**

	Inclusion (consultation avec le chirurgien) **T0**	**T1**	Phase de modélisation / Impression 3D	Consultation d échange et d'information **T2**	veille de l'intervention chirurgicale **T3**	Chirurgie **T4**	Période post-opératoire **T5**
		T0+1jr	T2-15jrs*	J-21 à J-4	J-1	J0	J+15
Information et recueil du consentement (R)	✓						
Randomisation (R)	✓						
Modélisation 3D (R) (selon bras randomisé)			✓				
Impression 3D (R) (selon bras randomisé)			✓				
Informations préopératoires (R) avec utilisation des modèles ou non selon le bras randomisé				✓			
Chirurgie (S)						✓	
Questionnaire STAI-Etat – *mesure de l'anxiété état* – 8' (R)		✓**			✓		✓
Questionnaire STAI-Trait - *mesure de l'anxiété trait* – 8' (R)		✓**					
EQ-5D-5L – *mesure de la qualité de vie* – 5' (R)		✓**					✓
HLSEU-Q16 – *mesure du niveau de littératie en santé* – 10' (R)		✓**					✓
Questionnaire de Wake –*mesure de la connaissance de la pathologie* – 5' (R)					✓		

(R) : recherche
(S) : soins courants
* 15 jours devant être pris en compte pour l'impression et la transmission au centre du modèle 3D imprimé.
** Le STAI-Y (état puis trait) sera demandée le lendemain de la visite d'inclusion. Un rappel sur UroCONNECT sera mis en place ou un rappel téléphonique pour les personnes ayant une version papier du questionnaire.

8.3. VISITE D'INCLUSION

8.3.1. Recueil du consentement

Lors de la visite d'inclusion, l'investigateur informe le participant et répond à toutes ses questions concernant l'objectif, la nature des contraintes, les risques prévisibles et les bénéfices attendus de la recherche. Il précise également les droits du participant dans le cadre d'une recherche impliquant la personne humaine et vérifie les critères d'éligibilité.

Un exemplaire de chaque note d'information (Rein-3D ANXIETY et UroCCR) est alors remis au participant par l'investigateur.

Après cette séance d'information, le participant dispose d'un délai de réflexion. L'investigateur est responsable de l'obtention des consentements éclairés auprès du participant. Les formulaires de consentement doit être signé AVANT LA REALISATION DE TOUT EXAMEN CLINIQUE OU PARACLINIQUE NECESSITE PAR LA RECHERCHE.

Si le participant donne son accord de participation, ce dernier et l'investigateur inscrivent leurs noms et prénoms en clair, datent et signent les deux formulaires des consentements en deux exemplaires originaux (un consentement pour la participation au projet Rein-3D ANXIETY et un consentement permettant de collecter les données nécessaires à l'analyse dans la base de données UroCCR) (annexe 6).

Un exemplaire original de chaque note d'information et de chaque consentement signé sera remis au participant.

Les autres exemplaires originaux seront conservés par l'investigateur (même en cas de déménagement du participant pendant la durée de la recherche) dans un lieu sûr, inaccessible à des tiers.

8.3.2. Deroulement de la visite

La visite d'inclusion est assurée par l'investigateur. Avant tout examen lié à la recherche, l'investigateur recueille le consentement libre et éclairé du participant (ou de son représentant légal le cas échéant).
A l'issue de cette entrevue, la totalité des patients est invitée, par l'équipe investigatrice, à remplir, soit sur l'interface UroConnect soit sur formulaire papier si pas d'accès à l'interface :

- Le lendemain de la visite d'inclusion :
  - Le STAI- Etat puis le STAI-Trait
  - Le EQ-5D-5L – questionnaire de qualité de vie
  - Le HLSEU-Q16 : European Health Literacy Survey – questionnaire de littératie

8.4. DEMARCHE DE RANDOMISATION

8.4.1. RANDOMISATION

La randomisation sera réalisée juste après la visite d'inclusion (et avant le remplissage des questionnaire du lendemain de la visite d'inclusion).

La randomisation se fera directement sur l'interface web de la base de données UroCCR.

Lorsqu'un investigateur souhaite effectuer la randomisation, après avoir vérifié l'éligibilité du participant, il se connecte avec ses codes sur le site Internet: www.uroccr.fr.
L'investigateur complète l'onglet « Recherche / Rein-3D ANXIETY» il confirme tous les critères d'éligibilité du participant. Après validation du contenu, l'inclusion et la randomisation sont effectuées. L'interface communique immédiatement à l'investigateur le numéro unique du participant dans la recherche et le résultat de la randomisation. **Le résultat de la randomisation ne sera communiqué au patient qu'à la visite d'échange et information pré-opératoire (T2).**

8.5. VISITES DE SUIVI

8.5.1. Consultation d'echange et information preoperatoire d'information (T2)

Les patients sont vus entre J-21 et J-4 de l'opération par un membre de l'équipe chirurgicale. Ce temps d'échange a pour vocation de permettre au patient d'exprimer ses interrogations, faire part de ses doutes ou incompréhensions en lien avec sa situation médicale et l'intervention programmée. Elle pourra déboucher sur la délivrance de commentaires anatomiques et de stratégie chirurgicale simples par le membre de l'équipe chirurgicale participant à cette entrevue.

Les deux groupes interventionnels (Groupe Modèle 3D Virtuel et Groupe Modèle 3D Imprimé) se voient présenter le modèle personnalisé de leur rein comme support de discussion.
Le Groupe contrôle bénéficie de la même entrevue pré-opératoire mais avec la fiche Info-Patient de l'AFU (qui présente des schémas génériques) utilisée comme support de discussion.
Les commentaires dont les patients bénéficient ne sont pas standardisés dans le cadre de l'étude afin de se rapprocher de la pratique quotidienne.
La durée de cette visite ainsi que le fait le patient soit accompagné ou non seront recueillis.

8.5.2. Veille de l'intervention chirurgicale (T3)

A J-1 de l'intervention chirurgicale la totalité des patients inclus est amenée à remplir via l'interface UroConnect (ou par formulaire papier en l'absence d'accès à UroConnect) :

- Le questionnaire STAI-Etat
- Le questionnaire d'évaluation de la compréhension de la maladie et du choix thérapeutique (traduction du questionnaire de Wake)

8.5.3. Chirurgie (T4)

Sans spécificité pour l'essai, mais comme pour tout patient inclus dans UroCCR, les caractéristiques et le déroulé technique de l'intervention chirurgicale seront collectés. Il n'y aura, du fait de l'essai, aucune modification de la technique chirurgicale de néphrectomie partielle robot-assistée telle qu'envisagée par le chirurgien.

8.6. VISITE DE FIN DE LA RECHERCHE

La recherche se terminera 15 jours après l'opération avec la complétion des derniers questionnaires par le patient participant à l'étude via UroConnect ou en format papier. Les questionnaires concernés sont :

- Le questionnaire STAI-Etat,
- Le EQ-5D-5L – questionnaire de qualité de vie,
- Le HLSEU-Q16 : European Health Literacy Survey – questionnaire de littératie.

Le patient sera revu dans les 3 mois après l'opération dans le cadre des visites classiques de suivi post-opératoire, cependant cette visite est hors recherche.

8.7. ABANDON ET RETRAIT DE CONSENTEMENT

Le participant qui souhaite abandonner ou retirer son consentement de participation à la recherche (comme il est en droit de le faire à tout moment) n'est plus suivi dans le cadre du protocole, mais doit faire l'objet de la meilleure prise en charge possible compte tenu de son état de santé et de l'état des connaissances du moment.

Un **abandon** est une décision d'un participant inclus de faire valoir son droit d'interrompre sa participation à une recherche, à tout moment au cours du suivi, sans qu'il n'encoure aucun préjudice de ce fait et sans avoir à se justifier.

Un **retrait de consentement** est une décision d'un participant de revenir sur sa décision de participer à une recherche et de faire valoir son droit d'annuler son consentement éclairé, à tout moment au cours du suivi et sans qu'il n'encoure aucun préjudice de ce fait et sans avoir à se justifier.

L'investigateur doit identifier la cause de l'abandon/du retrait et évalue s'il est possible de recueillir la variable sur laquelle porte le critère de jugement principal au moment de l'abandon/du retrait. Les abandons/retraits doivent être notifiés rapidement au centre investigateur coordonnateur, au promoteur et au centre de méthodologie et de gestion des données. Les raisons et la date d'abandon/de retrait doivent être documentées dans le cahier d'observation et dans le dossier médical du participant.

8.8. REGLES D'ARRET DE LA RECHERCHE

Fin de la recherche ou arrêt prévu de la recherche : terme de la participation de la dernière personne qui se prête à la recherche aussi appelé dernière visite du dernier participant inclus dans la recherche (toute autre définition doit être mentionnée dans le protocole).

Arrêt anticipé de la recherche : la recherche clinique est arrêté (définitivement) de façon anticipée.

8.9. DEVIATIONS AU PROTOCOLE

Les déviations peuvent concerner tous les aspects d'un protocole de recherche : processus d'inclusion, suivi, mesure des critères de jugement. Toutes doivent être documentées par l'investigateur et discutées en Conseil Scientifique.

Seuls les abandons et retrait de consentement entraînent un arrêt du suivi. Même en cas de déviation au protocole, le suivi du participant doit être mené jusqu'au terme prévu dans le protocole.

8.9.1. ARRET PREMATURE ET DEFINITIF DE LA STRATEGIE DE LA RECHERCHE

Un participant est considéré en arrêt de stratégie quand il l'arrête avant la date prévue dans le protocole. Les participants en arrêt prématuré continuent à être suivis comme prévu par le protocole.

Le participant qui arrête la stratégie fait l'objet de la meilleure prise en charge possible compte tenu de son état de santé et de l'état des connaissances du moment.

8.9.2. PARTICIPANT PERDU DE VUE

Les patients perdus de vue sont uniquement les patients qui ne se présentent pas en hospitalisation la veille de l'intervention chirurgicale, le recueil des données ne peut donc plus être effectué comme prévu.

8.9.3. PARTICIPANT INCLUS A TORT

Un participant est considéré comme inclus à tort lorsqu'il a effectivement été inclus dans la recherche alors qu'il ne vérifiait pas tous les critères d'éligibilité. Les participants inclus à tort font l'objet d'une discussion en Conseil Scientifique. Ils continuent à être suivis comme prévu par le protocole jusqu'à ce qu'une décision soit prise par le Conseil Scientifique.

8.10. RISQUES ET CONTRAINTES MINIMES LIEES A LA RECHERCHE ET INDEMNISATION EVENTUELLE DES PARTICIPANTS

La nature de l'essai Rein-3D ANXIETY, n'entraîne aucune modification des indications et stratégies de prise en charge clinique du patient, telles que validées par l'équipe chirurgicale selon les recommandations en vigueur et la conclusion de la réunion de concertation pluridisciplinaire (RCP).

9. GESTION DES ÉVÉNEMENTS INDÉSIRABLES / EFFETS INDESIRABLES /INCIDENTS

Les évènements indésirables / effets indésirables / incidents seront à déclarer aux différents circuits de vigilances sanitaires applicables à chaque produit ou pratique concernée (vigilance du soin, pharmacovigilance, matériovigilance, hémovigilance, cosmétovigilance…) en conformité avec la règlementation en vigueur.

Les déclarants doivent spécifier que le patient est inclus dans un essai clinique et identifier précisément l'essai clinique concerné.

10. ASPECTS STATISTIQUES

10.1. CALCUL DE LA TAILLE D'ETUDE

L'objectif de cette étude est d'évaluer l'effet de l'utilisation d'un modèle tridimensionnel personnalisé du rein du patient, lors d'une visite pré-opératoire d'information, sur l'anxiété pré-opératoire, en comparant un groupe avec modèle virtuel et un groupe avec modèle imprimé à un groupe sans modèle (groupe contrôle).
Nous faisons l'hypothèse que, dans le groupe contrôle, le niveau moyen d'anxiété pré-opératoires par STAI-Etat est de 55 (données d'une étude pilote, CHU de Bordeaux, non publiée) et qu'une différence cliniquement pertinente en faveur d'une des interventions évaluées est une réduction du score de 5 points (Biddiss E et al, Anesth Analg 2014). Avec un écart-type commun de 10 (étude pilote, CHU de Bordeaux), une puissance statistique de 80%, une erreur de type I bilatérale de 2,5 % (en tenant compte de 2 comparaisons avec le groupe contrôle dans cet essai à 3 bras, correction de Bonferroni), il faut inclure 78 patients par groupe, soit 234 patients au total (SAS® version n°9.4, « Proc power » avec un t-test bilatéral sur la différence de moyenne).

10.2. METHODES STATISTIQUES EMPLOYEES

10.1.1 STRATEGIE D'ANALYSE

Les données seront analysées par le biostatisticien du Centre de Méthodologie et de Gestion des données du CHU de Bordeaux (USMR).

L'analyse principale sera effectuée en intention-de-traiter (ITT), c'est à dire que tous les patients randomisés seront inclus dans l'analyse dans le groupe dans lequel ils ont été initialement randomisés, en utilisant la stratégie de gestion des données manquantes détaillée ci-dessous et que toutes leurs données seront utilisées quels que soient leurs changements de stratégie au cours de la recherche. Une analyse secondaire sera ensuite réalisée sur la population per-protocole où seules les données des participants ayant des données disponibles et ayant réellement eu une intervention chirurgicale par néphrectomie partielle coelioscopique avec assistance robotique pour tumeur du rein, seront analysées. Des analyses de sensibilité aux données manquantes pourront également être envisagées (exemple : analyse sur données disponibles avec prise en compte des seuls patients dont le critère considéré est renseigné).

Les objectifs secondaires seront analysés sur données disponibles. Des analyses de sensibilité aux données manquantes pourront également être envisagées (exemple : analyse par imputation multiple).
Une analyse descriptive sera réalisée de manière globale et par groupe de stratégie.
Une analyse de comparaison entre les groupes de randomisation sera réalisée, systématiquement :

- sans ajustement ;
- avec ajustement sur le facteur de stratification de la randomisation (le centre) et les facteurs pronostiques initiaux (notamment les scores STAI Trait et Etat à l'inclusion) dont la répartition pourrait être, malgré la randomisation, déséquilibrée entre les groupes de stratégie. Ces ajustements nécessiteront éventuellement d'utiliser des modèles appropriés dont la pertinence du choix sera discutée en fonction de la distribution et du type de variables.

10.1.2 STRATEGIE DE GESTION DES DONNEES MANQUANTES POUR LE CJP

Le STAI-Etat est un score additif, c'est-à-dire que pour obtenir le score, la somme des items correspondants est effectuée (codage entre 1 et 4 selon la modalité choisie), en prenant soin de recoder préalablement les items inversés). Les scores vont de 20 à 80, 20 indiquant le degré d'anxiété le plus faible et 80 le plus fort. La gestion des données manquantes pour des items isolées sera faite selon le manuel des scores (exemple : en cas d'absence de réponse à un item, le score moyen sans cet item est calculé en prenant pour dénominateur le nombre d'items renseignés); et par imputation multiple si le score global est manquant ou incalculable, à l'aide de la stratégie PROC-MI (SAS) et stratifiée sur le groupe de randomisation.
Il en sera de même pour la gestion des données manquantes des CJS correspondants à des scores. Elles seront faites selon le manuel des scores; et par imputation multiple si le score global est manquant ou incalculable.

Stratégies de gestion des données manquantes par imputation multiple : plusieurs jeux de données sont créés en remplaçant les données manquantes par des valeurs possibles, respectant les caractéristiques de la distribution observée. Les analyses sont réalisées sur chacun des jeux de données séparément puis les résultats sont combinés selon les règles de Rubin afin d'obtenir un résultat global.

10.1.3 Patients inclus dans l'analyse

Ne pourront être exclus de l'analyse que les patients qui présentent au moins une des conditions suivantes :

- patients inclus à tort pour consentement non signé ;
- patients inclus à tort pour critère(s) majeur(s) d'éligibilité non respecté(s) ;
- patients ayant retiré leur consentement.

Cette décision d'exclusion sera prise par le Conseil Scientifique en insu du groupe de stratégie et de l'évolution du patient après l'inclusion.
En dehors de ces exclusions, les patients décédés, perdus de vue ou ayant abandonné la recherche seront tous inclus dans l'analyse en ITT.

10.1.4 Risque de premiere espece

L'analyse principale sera effectuée au risque d'erreur global $\alpha = 5\%$ soit un seuil de p-valeur à 2.5% (correction de Bonferroni) pour chacune des deux comparaisons (Groupe 1 vs Groupe 3, Groupe 2 vs Groupe 3). Un ajustement du risque alpha pourra être envisagé pour l'analyse des objectifs secondaires.

10.1.5 Methodes statistiques descriptives

Le nombre ainsi que le pourcentage de participants avec des données manquantes sera décrit pour chaque variable d'intérêt. La raison des données manquantes sera documentée autant que possible afin d'interpréter les résultats.
Les variables qualitatives seront décrites en termes d'effectif, de pourcentage et d'intervalle de confiance à 95% selon la loi binomiale exacte.
Les variables quantitatives seront décrites en termes d'effectif, moyenne, écart-type, médiane, étendue et étendue interquartile.
On essayera autant que possible d'associer une représentation graphique aux analyses.

10.1.6 Methodes statistiques comparatives

Les distributions des variables qualitatives seront comparées entre les groupes par des tests du $\chi 2$, ou du $\chi 2$ corrigé, ou de Fisher exact, selon les valeurs des effectifs attendus sous l'hypothèse d'indépendance.
Un modèle de régression logistique ou polytomique sera utilisé afin de prendre en compte des ajustements si nécessaire. L'hypothèse de log-linéarité de l'association sera systématiquement vérifiée.
Les distribution des variables quantitatives seront comparées entre les groupes par le test de Student si les conditions de validité du test sont respectées (distribution normale, variances homogènes). Si les variances sont inégales entre les deux groupes, on utilisera un test de Student pour variances inégales, si la distribution n'est pas normale, on utilisera un test de Wilcoxon.
Un modèle de régression linéaire sera utilisé afin de prendre en compte les variables d'ajustement si nécessaire. L'hypothèse de linéarité de l'association sera systématiquement vérifiée. Les conditions d'application du modèle (normalité et homoscédasticité des résidus) seront étudiées. Une transformation de la variable pourra être envisagée si nécessaire.
Pour étudier l'évolution longitudinale des variables au cours du suivi, des modèles linéaires mixtes de données longitudinales seront utilisés

10.1.7 Logiciels utilises

Les analyses seront réalisées avec le logiciel SAS® (version 9.4 ou ultérieures).

10.2 PLAN D'ANALYSE

Un plan d'analyse statistique détaillé sera défini et fera l'objet d'une validation par le Conseil Scientifique de l'étude avant le gel de la base de données et les premières analyses. Les modifications ultérieures au gel de base de données seront systématiquement validées par le Conseil Scientifique et feront l'objet d'analyses post-hoc supplémentaires.

10.3.1 Description des inclusions, des deviations et du suivi

Les éléments suivants seront présentés :

- Vérification des critères d'éligibilité

- Le diagramme de flux de l'étude suivant les recommandations CONSORT
- Une description des causes de décès, d'abandon, des patients perdus de vue ou ayant abandonné la recherche, des patients n'ayant pas été opérés
- Les déviations au protocole (concernant notamment les délais)
- Visites de suivi réalisées : nombre de patients ayant réalisé chacune des visites de suivi

10.3.2 Caracteristiques des patients a l'inclusion

Les patients seront décrits selon les variables suivantes :

- caractéristiques épidémiologiques ;
- caractéristiques cliniques ;
- caractéristiques biologiques ;
- caractéristiques de la stratégie ;

10.3.3 Analyse de l'objectif principal

Le critère de jugement principal est le score moyen d'anxiété pré-opératoire par bras, mesuré à J-1 de la chirurgie par autoquestionnaire STAI-Etat.

Le STAI-Etat pré-opératoire sera décrit globalement et par groupes de procédure, en utilisant les méthodes statistiques descriptives détaillées précédemment (descriptif en termes d'effectif, moyenne, écart-type, médiane, étendue et étendue interquartile).

L'analyse principale sera effectuée au risque d'erreur global $\alpha = 5\%$ soit un seuil de p-valeur à 2.5% (correction de Bonferroni) pour chacune des deux comparaisons (« groupe Modèle 3D Virtuel vs Groupe contrôle » et « groupe Modèle 3D imprimé vs Groupe contrôle »), sans ajustement (test de Student ou de Wilcoxon).
Nous réaliserons ensuite un modèle de régression linéaire à effets mixtes pour estimer les différences entre les bras sur le score STAI-Etat pré-opératoire, en prenant en compte également sa valeur à l'inclusion, et en ajustant sur le centre (facteur de stratification de la randomisation), le score STAI-Trait à l'inclusion avec des termes d'interactions adéquats. Nous réaliserons pour cela des tests de comparaison spécifiques à T3 (à J-1) en utilisant les mesures STAI-Etat prédites par le modèle longitudinal.
Des analyses de médiation pourront être réalisées comme analyses complémentaires pour une meilleures compréhension du phénomène d'anxiété et seront définies dans le plan d'analyse statistique.

10.3.4 Analyse des objectifs secondaires

Les critères de jugement secondaires seront décrit globalement et par groupe de prise en charge selon les méthodes statistiques descriptives décrites au paragraphe précedemment (en terme d'effectif, moyenne, écart-type, médiane, minimum, maximum, 1er et 3ème quartile). Pour les critères de jugements secondaires associés à des évolutions (CJS 1, 2 et 4), les scores seront décrits à chaque temps de mesure puis les évolutions seront décrites selon la formule suivante : Delta = (Score à « T3 ou T5 » – score à T1). Nous décrirons également les scores par catégories (quand des catégories sont connues), globalement et par groupe de prise en charge à chaque temps de mesure, selon les méthodes statistiques descriptives décrites précedemment (en termes d'effectif, de pourcentage et d'intervalle de confiance à 95% selon la loi binomiale exacte) :

- STAI-Etat : inférieur ou égal à 35 (très faible), de 36 à 45 (faible), de 46 à 55 (moyen), de 56 à 65 (élevé) et supérieur à 65 (très élevé).
- HLSEU-Q16 inférieur ou égal à 8 traduisant un niveau inadéquat, de 9 à 12 traduisant un niveau problématique et strictement supérieur à 12 un niveau adéquat.

L'analyse comparative des critères de jugement secondaires (1, 2, 3 et 4) utilisera les méthodes statistiques comparatives détaillées précédemment :

- Pour le CJS n°1 (évolution du STAI-Etat) :
    - Un modèle de régression linéaire mixte sera réalisé en utilisant le score STAI-Etat de toutes les mesures (T1=inclusion, pré-opératoire=T3 et post-opératoire=T5). Des tests de comparaison spécifiques seront effectués à T3 (J-1) et T5 (J+15) en utilisant les mesures STAI-Etat prédites par le modèle longitudinal. Les mêmes variables d'ajustement que celles mentionnées pour

l'analyse du critère de jugement principal seront appliquées au modèle avec des termes d'interaction adéquates pour estimer les différence entre les bras sur l'évolution du STAI-Etat.

- Pour les CJS n°2 (évolution du HLSEU-Q16 à T1 et à T5) et n°4 (évolution du EQ-5D-5L à T1 et à T5), des modèles de régression linéaire à effets mixes pour les variables quantitatives seront utilisés pour estimer l'évolution du score entre T1 et T5et où les variables explicatives seront : le bras de randomisation, le centre (facteur de stratification de la randomisation) et les termes d'interaction adéquates.
- Pour le CJS n°3 (score Wake pré-opératoire à T3), un modèle de régression linéaire sera utilisé, où la variable à expliquer sera le score Wake pré-opératoire et où les variables explicatives seront : le bras de randomisation, le centre (facteur de stratification de la randomisation)
- Les hypothèses associées aux modèles seront systématiquement vérifiées.

11. SURVEILLANCE DE LA RECHERCHE

11.1. CONSEIL SCIENTIFIQUE

11.1.1 COMPOSITION

Il est composé des personnes suivantes : Pr Jean-Christophe BERNHARD (investigateur coordonnateur), Pr Laura RICHERT (Méthodologiste), Roxane COUERON (Biostatisticienne), Marthe-Aline Jutand, (chercheuse en sciences de l'éducation), Hélène Hoarau (anthropologue), Sarah Masanet, (représentant les disciplines sciences de l'éducation et de la formation et psychologie), Solène RICARD (Cheffe de Projet), Clémence MORICE (ARC coordinatrice), et un représentant du promoteur.

11.1.2 RYTHME DES REUNIONS

Le Conseil Scientifique de la recherche se réunit une fois par an.

11.1.3 ROLE

- Il a pour mission de prendre toute décision importante à la demande de l'investigateur coordonnateur concernant la bonne marche de la recherche et le respect du protocole.
- Il vérifie le respect de l'éthique.
- Il s'informe auprès du Centre de Méthodologie et de Gestion des données et du centre investigateur coordonnateur de la recherche de l'état d'avancement de la recherche, des problèmes éventuels et des résultats disponibles.
- Il décide de toute modification pertinente du protocole nécessaire à la poursuite de la recherche, notamment :
  - les mesures permettant de faciliter le recrutement dans la recherche,
  - les modifications des documents de la recherche (protocole et des documents d'information et de recueil de consentement)avant leur présentation au CPP,
  - les décisions d'ouvrir ou de fermer des sites participant à la recherche,
  - les mesures qui assurent aux personnes participant à la recherche la meilleure sécurité,
  - la discussion des résultats et la stratégie de publication de ces résultats.
- Le Conseil Scientifique peut proposer de prolonger ou d'interrompre la recherche en cas de rythme d'inclusion trop lent, d'un trop grand nombre de perdus de vue, de violations majeures du protocole ou bien pour des raisons médicales et/ou administratives. Il précise les modalités éventuelles du suivi prolongé des participants inclus dans la recherche.
- Les décisions concernant une modification majeure ou une modification de budget doivent être approuvées par le promoteur.

11.2. COMITE INDEPENDANT DE SURVEILLANCE

Cette étude ne nécessite pas la mise en place d'un comité indépendant de surveillance du fait de l'absence de tout traitement pouvant entraîner un arrêt prématuré de la recherche et la nature des procédures à l'étude n'entrainant aucun risque notable pour les patients.

12. DROITS D'ACCES AUX DONNEES ET DOCUMENTS SOURCE

12.1. ACCES AUX DONNEES

L'acceptation de la participation au protocole implique que les investigateurs mettront à disposition les documents et données individuelles strictement nécessaires au suivi, au contrôle qualité et à l'audit de la recherche, à la disposition des personnes ayant un accès à ces documents conformément aux dispositions législatives et réglementaires en vigueur.

12.2. DONNEES SOURCES

Ensemble des informations figurant dans des documents originaux, ou dans des copies authentifiées de ces documents, relatif aux examens cliniques, aux observations ou à d'autres activités menées dans le cadre d'une recherche et nécessaires à la reconstitution et à l'évaluation de la recherche. Les documents dans lesquels les données sources sont enregistrées sont appelés les documents sources.

12.3. CONFIDENTIALITE DES DONNEES

Conformément aux dispositions législatives en vigueur, les personnes ayant un accès direct aux données source prendront toutes les précautions nécessaires en vue d'assurer la confidentialité des informations relatives aux médicaments expérimentaux, aux recherches, aux personnes qui s'y prêtent et notamment en ce qui concerne leur identité ainsi qu'aux résultats obtenus. Ces personnes, au même titre que les investigateurs eux-mêmes, sont soumises au secret professionnel.

Pendant la recherche ou à son issue, les données recueillies sur les personnes qui s'y prêtent et transmises au promoteur par les investigateurs (ou tout autre intervenant spécialisé) seront rendues anonymes. Elles ne doivent en aucun cas faire apparaître en clair les noms des personnes concernées ni leur adresse.

Chaque participant se verra attribuer un code confidentiel d'identification, composé du numéro de centre investigateur (1 chiffres) et d'un numéro de participant (3 chiffres) et d'un code lettre (4 lettres) anonyme.

Le promoteur s'assurera que chaque personne qui se prête à la recherche a donné son consentement pour l'accès aux données individuelles la concernant et strictement nécessaires au contrôle de qualité de la recherche.

13. CONTROLE ET ASSURANCE QUALITE

13.1. CONSIGNES POUR LE RECUEIL DES DONNEES

Toutes les informations requises par le protocole doivent être consignées dans le dossier médical. Les données devront être recueillies au fur et à mesure qu'elles sont obtenues, et transcrites de façon nette et lisible. Les données médicales seront collectées dans la base UroCCR.

13.2. CONTROLE QUALITE

Un attaché de recherche clinique mandaté par le promoteur visite de façon régulière chaque centre investigateur, lors de la mise en place de la recherche, une ou plusieurs fois en cours de recherche selon le rythme des inclusions et en fin de recherche. Lors de ces visites, et conformément au plan de monitorage, les éléments suivants seront revus :

- ➢ consentement éclairé,
- ➢ respect du protocole de la recherche et des procédures qui y sont définies,

- qualité des données recueillies dans le cahier d'observation : complétude, exactitude, données manquantes, cohérence des données avec les documents source (dossiers médicaux, carnets de rendez-vous, originaux des résultats de laboratoire, etc,…),
- gestion des produits éventuels.

Toute visite fera l'objet d'un rapport de monitorage par compte-rendu écrit.

13.3. GESTION DES DONNEES

13.3.1 LOGICIEL DE GESTION DE DONNEES

13.3.1.1. LOGICIEL UTILISE

eCRF : Le logiciel utilisé pour la gestion des données est un eCRF accessible à l'adresse suivante : https://uroccr.fr
La maintenance et le développement informatiques de l'eCRF sont gérés par le CREDIM (Centre de Recherche et Développement en Informatique Médicale) qui est une plateforme informatique créée au sein de l'Université de Bordeaux.
UroConnect : La maintenance et le développement de l'application UroConnect est assurés par la société Resilience basée en France (https://www.resilience.care/).
Modélisation 3D : Le logiciel utilisé pour la modélisation 3D est Synapse 3D de Fujifilm.

13.3.1.2. HEBERGEMENT DES DONNEES

eCRF : Les données sont stockées sur un serveur dédié aux bases de données, géré par le CREDIM. Le système de gestion de base de données utilisé est Microsoft SQL server.
UroConnect : Les données collectées par UroConnect sont stockées dans un service hébergé en France (fournisseur Ecritel).
Modélisation 3D : Les modélisations 3D sont hebergés sur les serveurs ou sur l'ordinateur stand alone ayant le logiciel Synapse 3D de chaque centre participant à l'étude. Les modélisations pour l'impression 3D seront ensuite centralisées sur le serveur du CHU de Bordeaux (NextCloud) pour permettre un contrôle qualité. Une fois confirmé, les modélisation seront hebergées sur l'ordinateur du technicien du TechnoShop en charge de réaliser l'impression 3D.

13.3.1.3. SECURITE DES DONNEES

UroConnect : les données collectées par UroConnect sont stockées dans un environnemnet HDS.
eCRF : Le serveur se trouve dans une pièce dédiée, sans fenêtre. L'entrée dans le local sécurisé se fait au moyen d'un badge. Les portes du service sont sécurisées et fermées à clé le soir. Aucun ordinateur n'est en accès libre, l'authentification sur domaine est obligatoire.

La gestion des droits d'accès est gérée par le CREDIM pour les études du service.
Seuls les gestionnaires de la base, l'équipe projet et les auditeurs ont des droits d'accès direct à la base de données.

L'USMR transmettra au CREDIM la liste des personnes devant avoir accès aux données Les statisticiens et les DM auront donc accès aux données en lecture uniquement.

13.3.2 SAISIE DES DONNEES

UroCCR : La saisie des données est sous la responsabilité de l'investigateur du centre dans l'eCRF. Toute autre personne que l'investigateur effectuant la saisie dans l'eCRF doit être préalablement formée et déléguée par l'investigateur pour le faire.
Les questionnaires seront recueillis soit sur papier, soit directement via l'outil numérique UroCONNECT qui diffusera, sur un timing adapté, les différents questionnaires définis par le protocole par un envoi automatique aux patients.

13.3.3 CODAGE DES DONNEES

Les traitements prescrits et les événements cliniques sont codés dans l'eCRF afin de pouvoir effectuer le contrôle et l'analyse des données.
Les dictionnaires suivants sont utilisés pour le codage des termes médicaux :

- MedDRA (version en cours) FR/US
- ATC version

L'équipe investigatrice UroCCR est en charge du codage des données, sous responsabilité de l'investigateur.

13.3.4 CONTROLES DES DONNEES

Des contrôles sont programmés afin de vérifier la cohérence et la complétude des données saisies dans l'eCRF. La liste des contrôles à mettre en place est définie conjointement entre l'investigateur coordonnateur et l'USMR, dans le plan de validation des données de l'étude.
Le DM d'UroCCR et l'ARC coordonnateur est responsable de la gestion des demandes de correction, qu'il lance régulièrement.
L'investigateur fait les corrections nécessaires à la résolution des demandes de corrections.

13.3.5 TRANSFERT DES DONNEES

Les transferts de données (envoi, réception) sont réalisés conformément à la procédure en vigueur à l'USMR. Les modalités de transfert de données doivent être définies dans le Plan de Data Management. Pour des raisons de sécurité, les fichiers de données sont pseudonimisés puis transférés via la plateforme sécurisée CIRRUS ou NextCloud.
Dans le cadre de ce protocole :

- Les données pourront être transférées sur le serveur du CHU de Bordeaux (administré par la DSIN du CHU de Bordeaux) pour la réalisation des analyses statistiques par l'USMR.
- Un transfert des données est également prévue au laboratoire CeDS (Université de Bordeaux) pour des analyses complémentaires des données des auto-questionnaires.

D'autres transferts de données peuvent être demandées et approuvées selon la procédure en vigueur à l'USMR.

13.4. AUDIT ET INSPECTION

Sous réserve d'une information dans un délai de convenance, un audit peut être réalisé à tout moment par des personnes mandatées par le promoteur et indépendantes des personnes menant la recherche. Il a pour objectif de vérifier la sécurité des participants et le respect de leurs droits, le respect de la réglementation applicable et la fiabilité des données.

Une inspection pourrait également être diligentée par une autorité compétente..

L'audit, aussi bien que l'inspection, pourront s'appliquer à tous les stades de la recherche, du développement du protocole à la publication des résultats et au classement des données utilisées ou produites dans le cadre de la recherche.

Les investigateurs acceptent de se conformer aux exigences du promoteur en ce qui concerne un audit et à l'autorité compétente pour une inspection de la recherche.

14. CONSIDERATIONS ETHIQUES ET REGLEMENTAIRES

Le promoteur et l'(es) investigateur(s) s'engagent à ce que cette recherche soit réalisée en conformité avec la loi n°2012-300 du 5 mars 2012 relative aux recherches impliquant la personne humaine, ainsi qu'en accord avec les Bonnes Pratiques Cliniques (I.C.H. version 4 du 9 novembre 2016 et décision du 24 novembre 2006) et la déclaration d'Helsinki (qui peut être retrouvée dans sa version intégrale sur le site http://www.wma.net).

La recherche est conduite conformément au présent protocole. Hormis dans les situations d'urgence nécessitant la mise en place d'actes thérapeutiques précis, l'(es) investigateur(s) s'engage(nt) à respecter le protocole en tous points en particulier en ce qui concerne le recueil du consentement et la notification et le suivi des événements indésirables graves.

Cette recherche a reçu l'avis favorable du Comité de Protection des Personnes (CPP) de *nom du CPP* et a fait l'object d'une information auprès de l'ANSM.
Le CHU de Bordeaux, promoteur de cette recherche, a souscrit un contrat d'assurance en responsabilité civile auprès de Lloyd's Insurance Company SA (représentée par BEAH, mandataire) conformément aux dispositions du code de la santé publique.

Les données nécessaires à cette recherche sont enregistrées dans la base de données UroCCR qui a obtenu l'autorisation de la Commission Nationale de l'Informatique et des Libertés (CNIL) en date du 12/04/2013 (demande d'autorisation

n°912578, décision DR-2013-206). Les données de la base UroCCR font l'objet d'un traitement informatisé au CREDIM dans le respect de la loi n°78-17 du 6 janvier 1978 relative à l'informatique, aux fichiers et aux libertés modifiée par la loi 2004-801 du 6 août 2004.

Cette recherche entre dans le cadre de la « Méthodologie de référence » (MR-001) en application des dispositions de l'article 54 alinéa 5 de la loi du 6 janvier 1978 modifiée relative à l'information, aux fichiers et aux libertés. Ce changement a été homologué par décision du 5 janvier 2006, mise à jour le 21 juillet 2016. Le CHU de Bordeaux et l'USMR du CHU de Bordeaux a signé un engagement de conformité à cette « Méthodologie de référence ».

Cette recherche est enregistrée dans la base ID-RCB et est enregistrée sur le site http://clinicaltrials.gov/

MODIFICATIONS AU PROTOCOLE

Toute modification substantielle, c'est à dire toute modification de nature à avoir un impact significatif sur la protection des personnes, sur les conditions de validité et sur les résultats de la recherche, sur la qualité et la sécurité des produits expérimentés, sur l'interprétation des documents scientifiques qui viennent appuyer le déroulement de la recherche ou sur les modalités de conduite de celle-ci, fait l'objet d'un amendement écrit qui est soumis au promoteur ; celui-ci doit obtenir, préalablement à sa mise en œuvre, un avis favorable/autorisation des autorités concernées (CPP, CNIL, …).

Les modifications non substantielles, c'est à dire celles n'ayant pas d'impact significatif sur quelque aspect de la recherche que ce soit, sont communiquées au CPP à titre d'information.

Toutes les modifications sont validées par le promoteur, et par tous les intervenants de la recherche concernés, avant soumission au CPP. Cette validation peut nécessiter la réunion du CS.

Toutes les modifications au protocole doivent être portées à la connaissance de tous les investigateurs qui participent à la recherche. Les investigateurs s'engagent à en respecter le contenu.

Toute modification qui modifie la prise en charge des participants ou les bénéfices, risques et contraintes de la recherche fait l'objet d'une nouvelle note d'information et d'un nouveau formulaire de consentement dont le recueil suit la même procédure que celle précitée.

15. CONSERVATION DES DOCUMENTS ET DES DONNEES RELATIVES A LA RECHERCHE

Les documents suivants relatifs à cette recherche sont archivés conformément aux Bonnes Pratiques Cliniques et à la réglementation en vigueur :

- Le protocole et les modifications éventuelles au protocole
- Les cahiers d'observation (copies)
- Les dossiers source des participants ayant donné un consentement
- Tous les autres documents et courriers relatifs à la recherche
- L'exemplaire original des consentements éclairés signés des participants

Tous ces documents sont sous la responsabilité de l'investigateur pendant la durée réglementaire d'archivage.

Aucun déplacement ou destruction ne pourra être effectué sans l'accord du promoteur. Au terme de la durée réglementaire d'archivage, le promoteur sera consulté pour destruction. Toutes les données, tous les documents et rapports pourront faire l'objet d'audit ou d'inspection.

16. RAPPORT FINAL

Dans un délai d'un an suivant la fin de la recherche ou son interruption, un rapport final sera établi et signé par le promoteur et l'investigateur. Ce rapport sera tenu à la disposition de l'autorité compétente. Le promoteur transmettra à l'ANSM et au CPP les résultats de la recherche sous forme d'un résumé du rapport final dans un délai d'un an après la fin de la recherche.

17. REGLES RELATIVES A LA PUBLICATION

17.1. COMMUNICATIONS SCIENTIFIQUES

L'édition du rapport final est réalisé par l'USMR avec l'apport d'expertise du CeDS sur les questions méthodologiques autour des scores des questionnaires. . Cette analyse donne lieu à un rapport écrit qui est soumis au promoteur, qui transmettra au Comité de Protection des Personnes et à l'autorité compétente.

Toute communication écrite ou orale des résultats de la recherche doit recevoir l'accord préalable de l'investigateur coordonnateur et, le cas échéant, de tout comité constitué pour la recherche.

L'investigateur coordonnateur/principal s'engage à mettre à disposition du public les résultats de la recherche aussi bien négatifs et non concluants que positifs.

Pour la publication des résultats principaux, il faudra mentionner le nom du promoteur, de tous les investigateurs ayant inclus ou suivi des participants dans la recherche, des méthodologistes, biostatisticiens et data managers ayant participé à la recherche, des membres du(des) comité(s) constitué(s) pour la recherche et la mention « *ce travail a bénéficié d'une aide de l'Etat gérée par l'Agence Nationale de la Recherche au titre du troisième PIA intégré à France 2030 portant la référence ANR-21-RHUS-0015* ». Il sera tenu compte des règles internationales d'écriture et de publication (The Uniform Requirements for Manuscripts de l'ICMJE, avril 2010).

17.2. COMMUNICATION DES RESULTATS AUX PARTICIPANTS

Conformément à la loi n°2002-303 du 4 mars 2002, les participants sont informés, à leur demande, des résultats globaux de la recherche.

17.3. CESSION DES DONNEES

La gestion des données est assurée par le CHU de Bordeaux. Les conditions de cession de tout ou partie de la base de données de la recherche sont décidées par le promoteur de la recherche et font l'objet d'un contrat écrit.

REFERENCES BIBLIOGRAPHIQUES

1. Ruszniewski, M., Rabier, G. (2015). *L'Annonce: Dire la maladie grave*. Dunod, Paris.
2. Bernhard, J. C., Isotani, S., Matsugasumi, T., Duddalwar, V., Hung, A. J., Suer, E., ... & Hu, B. (2016). Personalized 3D printed model of kidney and tumor anatomy: a useful tool for patient education. World journal of urology, 34(3), 337-345
3. Biddiss, E., Knibbe, T. J., & McPherson, A. (2014). The effectiveness of interventions aimed at reducing anxiety in health care waiting spaces: a systematic review of randomized and nonrandomized trials. Anesthesia & Analgesia, 119(2), 433-448.
4. Spielberger, C. D., Gorsuch, R. L., Lushene, R., Vagg, P. R., & Jacobs, G. A. (1983). Manual for the State-Trait Anxiety Inventory (Form Y). Palo Alto, CA: Consulting Psychologists Press.
5. Spielberger, C. D., & Bruchon-Schweitzer, M. (1993). STAI-Y : Inventaire d'anxiété état-trait forme Y. Éditions du centre de psychologie appliquée.
6. Rouquette A, NadotT, LabitrieP, Van denBrouckeS, ManciniJ, RigalL, et al. (2018)Validityand measurement invarianceacrosssex, age, andeducationlevel of the Frenchshortversionsof theEuropeanHealthLiteracySurveyQuestionnaire.PLoSONE 13(12):e0208091. https://doi.org/10.1371/journal.pone.0208091
7. Sørensen, K., Slonska, Z., Kondilis, B., Stoffels, V., Osborne, R. H., & Brand, H. (2013). Measuring health literacy in populations : Illuminating the design and development process of the European Health Literacy Survey Questionnaire (HLS-EU-Q). 10.
8. Aaronson, N. K.,
9. Wake N, Rosenkrantz AB, Huang R, Park KU, Wysock JS, Taneja SS, Huang WC, Sodickson DK, Chandarana H. Patient-specific 3D printed and augmented reality kidney and prostate cancer models: impact on patient education. 3D Print Med. 2019 Feb 19;5(1):4. doi: 10.1186/s41205-019-0041-3. PMID: 30783869; PMCID: PMC6743040.
10. Herdman M, Gudex C, Lloyd A, Janssen M, Kind P, Parkin D, Bonsel G, Badia X. Development and preliminary testing of the new five-level version of EQ-5D (EQ-5D-5L). Qual Life Res. 2011 Dec;20(10):1727-36. doi: 10.1007/s11136-011-9903-x. Epub 2011 Apr 9. PMID: 21479777; PMCID: PMC3220807.
11. Andrade LF, Ludwig K, Goni JMR, Oppe M, de Pouvourville G. A French Value Set for the EQ-5D-5L. Pharmacoeconomics. 2020 Apr;38(4):413-425. doi: 10.1007/s40273-019-00876-4. PMID: 31912325; PMCID: PMC7080328.

ANNEXES

Annexe 1 : Fiche Info-Patient de l'AFU

FICHE INFO PATIENT

ASSOCIATION FRANÇAISE D'UROLOGIE

FICHE CRÉÉE EN : AVANT 2012
DERNIERE MISE A JOUR : NOVEMBRE 2018

FICHE REMISE LE …………/…………/…………

PAR DR …………………………………………………

Madame, Monsieur,
Cette fiche, rédigée par l'Association Française d'Urologie est un document destiné à vous aider à mieux comprendre les informations qui vous ont été expliquées par votre urologue à propos de votre maladie et des choix thérapeutiques que vous avez faits ensemble.
En aucune manière ce document ne peut remplacer la relation que vous avez avec votre urologue. Il est indispensable en cas d'incompréhension ou de question supplémentaire que vous le revoyiez pour avoir des éclaircissements.
Vous sont exposées ici les raisons de l'acte qui va être réalisé, son déroulement et les suites habituelles, les bénéfices et les risques connus même les complications rares.
Prenez le temps de lire ce document éventuellement avec vos proches ou votre médecin traitant, revoyez votre urologue si nécessaire. Ne vous faites pas opérer s'il persiste des doutes ou des interrogations.

Pour plus d'information, vous pouvez consulter le site : www.urologie-santé.fr

NÉPHRECTOMIE PARTIELLE / TUMORECTOMIE POUR TUMEUR

Votre urologue se tient à votre disposition pour tout renseignement.

Cette intervention est destinée à retirer la partie de votre rein qui est le siège d'une tumeur.

_ RAPPEL ANATOMIQUE

Le rein est un organe qui joue le rôle d'un filtre participant à l'épuration du sang et à l'élimination des déchets de l'organisme.
Les reins sont habituellement au nombre de deux. Ils sont situés dans l'abdomen sous le thorax, de part et d'autre de la colonne vertébrale. L'urine fabriquée par les reins est drainée par les uretères vers la vessie où elle est stockée entre deux mictions.
Un seul rein peut suffire à assurer cette fonction d'épuration.

_ POURQUOI CETTE INTERVENTION ?

Les examens demandés par votre médecin ont mis en évidence une tumeur du rein.
La taille de la tumeur et sa localisation permettent d'envisager de conserver une partie du rein ; ainsi, le traitement chirurgical qui vous est proposé consiste à enlever la partie du rein, où se trouve la tumeur.

Cette opération est nécessaire car, en l'absence de traitement, cette tumeur vous expose au risque de progression de la maladie tumorale (douleurs, saignements, fièvre, chimiothérapie, métastases, décès).

_ EXISTE-T-IL D'AUTRES POSSIBILITÉS ?

Certaines petites tumeurs peuvent être simplement surveillées.
Pour les autres, une alternative au traitement chirurgical existe. Il s'agit de techniques dites ablatives, qui consistent à détruire la tumeur par l'application de froid (cryothérapie) ou de chaleur (radiofréquence, micro-ondes). D'autres techniques par radiothérapie focalisée peuvent être proposées.
Cependant, ces techniques qui ne réalisent pas l'exérèse de la tumeur sont réservées pour des indications spécifiques.
Votre urologue vous a expliqué pourquoi il vous propose une néphrectomie partielle / tumorectomie.

_ PRÉPARATION À L'INTERVENTION

Toute intervention chirurgicale nécessite une préparation qui peut être variable selon chaque individu. Il est indispensable que vous suiviez les recommandations qui vous seront données par votre urologue et votre anesthésiste. En cas de non-respect de ces recommandations, l'intervention pourrait être reportée.

Avant chaque intervention chirurgicale, une consultation d'anesthésie pré-opératoire est obligatoire.

Il est impératif de signaler à votre urologue et à l'anesthésiste vos antécédents médicaux, chirurgicaux, allergiques et vos traitements en cours, en particulier anticoagulants oraux ou injectables ou antiagrégants (aspirine, clopidogrel, anti vitamine K…). Leur utilisation augmente le risque de saignement lors de l'intervention. Ce traitement pourra être adapté et éventuellement modifié avant l'intervention.

_ TECHNIQUE OPÉRATOIRE

L'intervention se déroule sous anesthésie générale.
Plusieurs voies d'abord permettent d'accéder au rein :

- Une incision classique antérieure ou sur le côté de l'abdomen (lombotomie)
- Une voie coelioscopique avec ou sans assistance robotique

Le choix est fait en fonction de la localisation de la tumeur, de votre morphologie, de vos antécédents et des habitudes de votre chirurgien.

Il est possible :

- Qu'une sonde soit mise en place dans l'uretère au cours de l'intervention,
- Que la voie d'abord soit modifiée pendant l'intervention en raison de difficultés.

Le geste chirurgical consiste à enlever la tumeur en préservant la partie saine du rein. Dans la plupart des cas, la circulation sanguine du rein doit être interrompue momentanément par un clampage (occlusion temporaire des vaisseaux du rein), le temps de l'exérèse de la tumeur.
En fin d'intervention, des drains peuvent être mis en place. Ils permettent de surveiller les écoulements par le site opératoire.
La pièce opératoire, retirée lors de l'intervention, est secondairement analysée au microscope lors de l'examen anatomopathologique. Cet examen est important pour décider des suites à donner à votre maladie : surveillance, reprise chirurgicale, chimiothérapie…

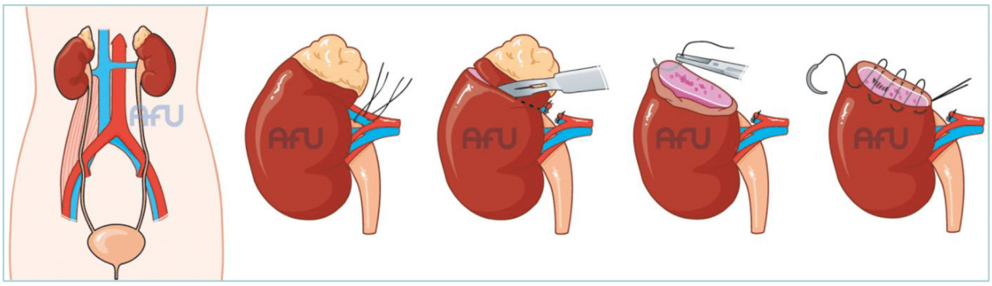

_ SUITES HABITUELLES

Pour surveiller le bon fonctionnement des reins et vous éviter d'avoir des difficultés urinaires au réveil, une sonde urinaire peut être mise en place pendant l'intervention. Cette sonde peut être responsable d'un inconfort.
La douleur post-opératoire est prise en charge par l'administration d'antalgiques. Un cathéter peut être placé dans la cicatrice pour diminuer les douleurs des premières heures.
Vous êtes habituellement autorisé à vous lever et à vous réalimenter de manière précoce.
L'ablation du ou des drains et de la sonde urinaire est définie par le chirurgien. Ces ablations ne sont qu'exceptionnellement douloureuses.
La durée d'hospitalisation est variable et une convalescence de quelques semaines est nécessaire.
Vous discuterez avec votre chirurgien de la date de reprise de vos activités et du suivi après l'opération.

_ RISQUES ET COMPLICATIONS

Dans la majorité des cas, l'intervention qui vous est proposée se déroule sans complication. Cependant, tout acte chirurgical comporte un certain nombre de risques et complications décrits ci-dessous.

Certaines complications sont liées à votre état général.

Toute intervention chirurgicale nécessite une anesthésie, qu'elle soit loco-régionale ou générale, qui comporte des risques. Elles vous seront expliquées lors de la consultation pré-opératoire avec le médecin anesthésiste.

D'autres complications directement en relation avec l'intervention sont rares, mais possibles.

LES COMPLICATIONS COMMUNES À TOUTE CHIRURGIE SONT :

- Infection locale, généralisée
- Le saignement avec hématome possible et parfois transfusion
- Phlébite et embolie pulmonaire
- Allergie

LES COMPLICATIONS DIRECTEMENT EN RELATION AVEC L'INTERVENTION SONT RARES VOIRE EXCEPTIONNELLES.

Elles sont listées ci-dessous par ordre de fréquence :

↘ Pendant le geste opératoire

- Blessure des organes de voisinage (foie, rate, tube digestif, glande surrénale, pancréas, diaphragme, nerfs…) pouvant nécessiter un geste complémentaire, un changement de voie d'abord, l'intervention d'autres chirurgiens. Les décès restent exceptionnels mais possibles.
- Blessure d'un vaisseau sanguin responsable d'un saignement pouvant nécessiter une transfusion de sang ou un geste de réparation vasculaire complémentaire.
- Conservation du rein impossible ou à risque : dans ce cas, votre chirurgien prend la décision de réaliser une néphrectomie totale, c'est-à-

dire **l'ablation du rein dans sa totalité**.

- Absence de revascularisation du rein lors de sa remise en circulation pouvant justifier l'ablation du rein en totalité.
- Blessure de l'uretère pouvant nécessiter la pose d'une sonde.
- Possibilité d'un geste plus complexe en raison de la découverte pendant l'intervention d'autres anomalies sur le rein (2ème tumeur) ou d'adhérences inhabituelles.
- Un changement de voie d'abord peut être nécessaire en fonction des conditions techniques (ouverture de l'abdomen).

↘ Dans les suites postopératoires précoces

- Risque d'infection (site opératoire, paroi, urines, poumon, site de perfusion) pouvant justifier un geste complémentaire radiologique ou chirurgical.
- Saignement pouvant nécessiter un geste complémentaire : embolisation (oblitération du vaisseau sanguin sous contrôle radiologique), une transfusion ou reprise chirurgicale.
- Problèmes cardio-vasculaires ou liés à l'anesthésie nécessitant une prise en charge dans un service de soins intensifs. Les causes les plus fréquentes sont les infections pulmonaires, les embolies pulmonaires, les accidents vasculaires cérébraux, les phlébites, les infarctus du myocarde dont les formes les plus sévères peuvent aboutir au décès.
- Risque de pneumothorax (diffusion d'air autour du poumon) pouvant justifier la mise en place d'un drain thoracique.
- Complications digestives :

  - Retard à la reprise du transit intestinal ou véritable occlusion.
  - infection abdominale par fistule digestive nécessitant une intervention chirurgicale en urgence.
  - Eventration ou éviscération nécessitant habituellement une ré-intervention et dans des cas exceptionnels, la réalisation d'une stomie digestive temporaire (anus artificiel).
  - Ulcère de l'estomac relevant le plus souvent d'un traitement médical prolongé.
- Oblitération secondaire de l'artère rénale responsable de la perte définitive du rein.
- Fistule urinaire due à une mauvaise cicatrisation de la voie excrétrice, à l'origine d'un écoulement d'urines par le drainage de paroi ou par la cicatrice. Parfois, votre chirurgien doit drainer la voie excrétrice par une sonde extériorisée ou par une sonde interne afin d'assécher la fistule. Néanmoins, en cas d'échec, une nouvelle intervention peut être indiquée pour refermer la voie excrétrice, voire pour enlever le rein. Des soins infirmiers de la cicatrice peuvent aussi être nécessaires pendant plusieurs semaines ou mois.
- Risque de fistule artério-veineuse nécessitant une ablation du rein en urgence. Cette complication est exceptionnelle mais potentiellement mortelle.
- En cas de néphrectomie totale sur rein unique, il peut arriver de faire appel à la dialyse.

↘ Risques à distance

- Comme dans toute intervention abdominale, des brides intra-abdominales peuvent survenir et entraîner des troubles digestifs.
- Des déformations de la paroi de l'abdomen au niveau de l'incision peuvent apparaître ou s'aggraver avec le temps. Il peut s'agir d'une déhiscence des muscles de l'abdomen, ou d'une hypotonie (diminution du tonus musculaire) séquellaire de l'intervention.
- L'insuffisance rénale chronique
- L'hypertension artérielle
- Des collections liquidiennes ou des abcès peuvent nécessiter un drainage plusieurs semaines après l'intervention.
- Des troubles de la sensibilité cutanée pouvant apparaître le long ou en dessous de la cicatrice.
- Des problèmes cutanés ou neurologiques liés à votre position sur la table d'opération ou à l'alitement prolongé pouvant entraîner des séquelles et une prise en charge à long terme.
- Le risque de recours provisoire ou définitif au rein artificiel (dialyse).
- La récidive de la maladie sur le site d'exérèse de la tumeur, dans une autre portion du rein opéré, dans les tissus de voisinage ou sur la cicatrice justifiant des traitements spécifiques.

_ SUITES D'INTERVENTION

INFORMATIONS GÉNÉRALES

Après l'intervention, il vous est conseillé d'éviter tout effort ou déplacement important dans le premier mois suivant l'intervention.

Les ordonnances remises à la sortie peuvent comprendre des soins ainsi que l'injection quotidienne d'un anti-coagulant. Le maintien d'un traitement anticoagulant est nécessaire après votre hospitalisation pour prévenir le risque de phlébite. Le port des bas de contention peut être souhaitable au moins 10 jours après l'intervention.

Un courrier est adressé à votre médecin traitant pour le tenir informé de votre état de santé.

La durée de la convalescence et la date de reprise du travail ou d'une activité physique normale dépendent de votre état physique et du geste réalisé. Vous discuterez avec votre urologue de la date de reprise de vos activités et du suivi après l'opération.

Une consultation post opératoire est programmée avec votre urologue afin de vous informer du résultat de l'examen microscopique de votre tumeur rénale.

PRÉCAUTIONS

↘ Prévention d'une phlébite et embolie pulmonaire

L'alitement et l'absence de mouvement des membres inférieurs favorisent la stase veineuse. Des douleurs dans une jambe, une sensation de pesanteur ou une diminution du ballotement du mollet doivent faire évoquer une phlébite. Il est donc nécessaire de consulter un médecin en urgence.

Afin d'éviter la survenue d'une phlébite, il est conseillé de suivre les recommandations : contractions régulières et fréquentes des mollets, mouvements des pieds, surélévation des jambes et suivant la prescription de votre médecin, port de bas de contention.
En cas de douleur thoracique, de point de coté, de toux ou d'essoufflement, il est nécessaire de consulter en urgence car ces signes peuvent être révélateurs d'une embolie pulmonaire. Contactez alors immédiatement votre médecin traitant ou votre urologue ou le service des urgences le plus proche en téléphonant au Centre 15.

↘ Cicatrisation

Les incisions sont des portes d'entrée possibles pour une infection. Il est donc nécessaire de s'assurer d'une bonne hygiène locale. Si une cicatrice devient rouge, chaude ou s'il existe une surélévation de celle-ci, il est important de montrer cette cicatrice à votre chirurgien ou votre médecin traitant.

La cicatrisation cutanée s'effectue en plusieurs jours. Durant cette période, il peut se produire un petit saignement que l'on peut stopper en le comprimant à l'aide d'une compresse ou d'un linge propre. Certains fils de suture sont résorbables et d'autres seront retirées par une infirmière suivant la prescription médicale de sortie.
Une désunion de la peau peut parfois survenir. Si cette ouverture est superficielle, il faut simplement attendre qu'elle se referme. Le délai de fermeture peut atteindre plusieurs semaines.

Le tabac et la dénutrition ralentissent la cicatrisation.

Les cicatrices ne doivent pas être exposées au soleil pendant 1 an.

↘ Des troubles du transit intestinal

Après une intervention abdominale, le retour au transit digestif parfaitement normal peut nécessiter quelques semaines. Des troubles du transit sont fréquents. Une période de plusieurs jours sans selle n'est pas un signe inquiétant. A l'opposé, l'absence de gaz, des nausées ou des vomissements nécessitent une consultation en urgence (risque d'occlusion).

Pour faciliter la reprise d'un transit normal, il est conseillé de :

- Manger de petites quantités à chaque repas en mastiquant lentement
- Prendre ses repas assis, dans le calme
- Arrêter de manger dès les premiers tiraillements digestifs

- Ne pas trop boire en mangeant, mais boire suffisamment entre les repas
- Manger équilibré et le plus varié possible pour éviter les carences nutritionnelles
- Respecter un apport suffisant en protéines (viandes, œufs, poissons, produits laitiers…)
- Eviter les abus de boissons gazeuses, les sauces et les fritures, ainsi que les sucreries et les aliments gras.

SIGNES QUI PEUVENT SURVENIR ET CONDUITE À TENIR

↘ Ces situations nécessitent une consultation URGENTE auprès de votre urologue ou de votre médecin traitant.

- Des sueurs, un essoufflement, des palpitations et / ou une pâleur cutanée
- Des urines très sanglantes
- Des douleurs intenses du côté opéré ou de l'abdomen
- Un écoulement abondant par la cicatrice

↘ Ces situations nécessitent une consultation RAPIDE auprès de votre urologue ou de votre médecin traitant.

- Une fatigue inhabituelle
- Des douleurs musculaires
- Une fièvre
- Des douleurs des épaules
- Des douleurs au niveau de la plaie
- Une sensation persistante de pesanteur du côté opéré, des troubles du transit intestinal ou de la fièvre

Il vous est recommandé de boire abondamment. Les urines peuvent contenir un peu de sang pendant quelques jours.

Questions pratiques

- Comment puis-je me laver ?

  Dès votre retour à domicile, vous pouvez prendre une douche.

- Puis-je faire du sport ?

  La reprise de vos activités est possible 1 à 3 mois après l'intervention.

- Puis-je conduire après l'intervention ?

  Certains médicaments contre les douleurs peuvent entrainer une somnolence qui peut ne pas être compatible avec la conduite. La conduite d'un véhicule personnel est possible selon l'accord de votre chirurgien.

- Puis-je voyager ?

  Les voyages sont possibles selon l'accord de votre chirurgien.

- Quand puis-je reprendre une activité sexuelle ?

  La reprise d'une activité sexuelle est possible dès que votre état général le permet.

Il est difficile de répondre ici à toute vos questions, n'hésitez pas à contacter votre urologue ou votre médecin traitant.

_ SUIVI POST-OPÉRATOIRE

Le suivi dont vous ferez l'objet après l'intervention répond à trois objectifs :

- Vérifier l'absence de récidive en cas de tumeur cancéreuse.
- Surveiller le fonctionnement des reins.
- Détecter d'éventuelles complications.

Lors de la consultation post-opératoire, votre urologue vous informe du résultat de l'analyse microscopique de la tumeur, du stade et du pronostic de votre maladie. L'ensemble de ces éléments conditionne la fréquence et les modalités de votre surveillance qui reposera sur une surveillance clinique associée à des examens biologiques et radiologiques.

Il est rappelé que **toute intervention chirurgicale comporte un certain nombre de risques y compris vitaux**, tenant à des variations individuelles qui ne sont pas toujours prévisibles. Certaines de ces complications sont de survenue exceptionnelle (plaies des vaisseaux, des nerfs et de l'appareil digestif) et peuvent parfois ne pas être guérissables. Au cours de cette intervention, le chirurgien peut se trouver en face d'une découverte ou d'un événement imprévu nécessitant des actes complémentaires ou différents de ceux initialement prévus, voire une interruption du protocole prévu.
Toute chirurgie nécessite une mise au repos et une diminution des activités physique. Il est indispensable de vous mettre au repos et de ne reprendre vos activités qu'après accord de votre chirurgien.

EN CAS D'URGENCE,
votre urologue vous donnera la conduite à tenir.

En cas de difficulté à le joindre,
faites le 15.

Fumer augmente le risque de complications chirurgicales de toute chirurgie, en particulier risque infectieux (X3) et difficulté de cicatrisation (X5). Arrêter de fumer 6 à 8 semaines avant l'intervention diminue significativement ces risques. De même, Il est expressément recommandé de ne pas recommencer à fumer durant la période de convalescence.

➘ Si vous fumez, parlez-en à votre médecin, votre chirurgien et votre anesthésiste ou appelez la ligne
Tabac-Info-Service au 3989
ou par internet :
tabac-info-Service.fr,
pour vous aider à arrêter.

CONSENTEMENT ÉCLAIRÉ

DOCUMENT DE CONSENTEMENT AUX SOINS

Dans le respect du code de santé publique (Article R.4127-36), je, soussigné (e) Monsieur, Madame, ……………………………………………… reconnaît avoir été informé (e) par le Dr …………………………………………… en date du ………/………/………, à propos de l'intervention qu'il me propose : **néphrectomie partielle.**

J'ai bien pris connaissance de ce document et j'ai pu interroger le Dr …………………………………………… qui a répondu à toutes mes interrogations et qui m'a rappelé que je pouvais jusqu'au dernier moment annuler l'intervention.

Ce document est important. Il est indispensable de le communiquer avant l'intervention. En son absence, votre intervention sera annulée ou décalée.

Fait à ………………………………………..

Le ………/………/………

En 2 exemplaires,

Signature

Cette fiche a été rédigée par l'Association Française d'Urologie pour vous accompagner. Elle ne doit pas être modifiée. Vous pouvez retrouver le document original et des documents d'information plus exhaustifs sur le site **urologie-santé.fr**
L'Association Française d'Urologie ne peut être tenue responsable en ce qui concerne les conséquences dommageables éventuelles pouvant résulter de l'exploitation des données extraites des documents sans son accord.

PERSONNE DE CONFIANCE

Madame, Monsieur,

En application de la loi du 4 mars 2002, dite « loi Kouchner » sur le droit des patients, il nous est demandé d'améliorer leur environnement proche lors de leur prise en charge.

En plus du consentement éclairé qui décrit l'indication et les risques de l'intervention que vous allez prochainement avoir, nous vous prions de trouver ci-joint une fiche de désignation d'une personne de confiance.
Cette désignation a pour objectif, si nécessaire, d'associer un proche aux choix thérapeutiques que pourraient être amenés à faire les médecins qui vous prendront en charge lors de votre séjour. C'est une assurance, pour vous, qu'un proche soit toujours associé au projet de soin qui vous sera proposé.
Elle participera aux prises de décisions de l'équipe médicale si votre état de santé ne vous permet pas de répondre aux choix thérapeutiques.

Nous vous remercions de bien vouloir remplir consciencieusement ce document et de le remettre à l'équipe soignante dès votre arrivée.

☐ JE NE SOUHAITE PAS DÉSIGNER UNE PERSONNE DE CONFIANCE

À ……………………………………………………………………

le ………/……………/…………..

Signature

☐ JE SOUHAITE DÉSIGNER UNE PERSONNE DE CONFIANCE

Cette personne est :

Nom : ………………………………………………………………………… Prénom : ………………………………………………………………

Lien (époux, épouse, enfant, ami, médecin….) : ………………………………………………………………………………………

Téléphone fixe : ……………………………………………….Téléphone portable : ………………………………………………………

Adresse : ………

………

J'ai été informé(e) que cette désignation vaut pour toute la durée de mon hospitalisation. Je peux révoquer cette désignation à tout moment et dans ce cas, je m'engage à en informer par écrit l'établissement en remplissant une nouvelle fiche de désignation.

Date de confiance :

………/……………/……………

Signature	*Signature de la personne*

Annexe 2 : ECHELLE STAI Etat

Un certain nombre de phrase que l'on utilise pour se décrire sont données ci-dessous. Lisez chaque phrase, puis entourez, parmi les 4 points à droite, celui correspond le mieux à ce que vous ressentez A L'INSTANT, JUSTE EN CE MOMENT. Il n'y a pas de bonnes ni de mauvaises réponses. Ne passez pas trop de temps sur l'une ou l'autre de ces propositions et indiquez la réponse qui décrit le mieux vos sentiments ACTUELS.

1.	Je me sens calme	Non	Plutôt non	Plutôt oui	Oui
2.	Je me sens en sécurité, sans inquiétude, en sûreté	Non	Plutôt non	Plutôt oui	Oui
3.	Je suis tendu(e), crispé(e)	Non	Plutôt non	Plutôt oui	Oui
4.	Je me sens surmené(e)	Non	Plutôt non	Plutôt oui	Oui
5.	Je sens tranquille, bien dans ma peau	Non	Plutôt non	Plutôt oui	Oui
6.	Je me sens ému(e), boulversé(e), contrarié(e)	Non	Plutôt non	Plutôt oui	Oui
7.	L'idée de malheurs éventuels me tracasse en ce moment	Non	Plutôt non	Plutôt oui	Oui
8.	Je me sens content(e)	Non	Plutôt non	Plutôt oui	Oui
9.	Je me sens effrayé(e)	Non	Plutôt non	Plutôt oui	Oui
10.	Je me sens à mon aise (je me sens bien)	Non	Plutôt non	Plutôt oui	Oui
11.	Je sens que j'ai confiance en moi	Non	Plutôt non	Plutôt oui	Oui
12.	Je me sens nerveux (nerveuse), irritable	Non	Plutôt non	Plutôt oui	Oui
13.	J'ai la frousse, la trouille (j'ai peur)	Non	Plutôt non	Plutôt oui	Oui
14.	Je me sens indécis(e)	Non	Plutôt non	Plutôt oui	Oui
15.	Je suis décontracté(e), détendu(e)	Non	Plutôt non	Plutôt oui	Oui
16.	Je suis satisfait(e)	Non	Plutôt non	Plutôt oui	Oui
17.	Je suis inquiet, soucieux (inquiète, soucieuse)	Non	Plutôt non	Plutôt oui	Oui
18.	Je ne sais plus où j'en suis, je me sens déconcerté(e), dérouté(e)	Non	Plutôt non	Plutôt oui	Oui
19.	Je me sens solide, posé(e), pondéré(e), réfléchi(e)	Non	Plutôt non	Plutôt oui	Oui
20.	Je me sens de bonne humeur, aimable	Non	Plutôt non	Plutôt oui	Oui

Validations : Spielberger, C. D., Gorsuch, R. L., Lushene, R., Vagg, P. R., & Jacobs, G. A. (1983). Manual for the State-Trait Anxiety Inventory (Form Y). Palo Alto, CA: Consulting Psychologists Press.
Spielberger, C. D., & Bruchon-Schweitzer, M. (1993). STAI-Y : Inventaire d'anxiété état-trait forme Y. Éditions du centre de psychologie appliquée.

Annexe 3 : ECHELLE STAI Trait

Un certain nombre de phrase que l'on utilise pour se décrire sont données ci-dessous. Lisez chaque phrase, puis entourez, parmi les 4 points à droite, celui correspond le mieux à ce que vous ressentez GENERALEMENT. Il n'y a pas de bonnes ni de mauvaises réponses. Ne passez pas trop de temps sur l'une ou l'autre de ces propositions et indiquez la réponse qui décrit le mieux vos sentiments HABITUELS.

1.	Je me sens de bonne humeur, aimable	Presque jamais	Parfois	Souvent	Presque toujours
2.	Je me sens nerveux (nerveuse) et agitée	Presque jamais	Parfois	Souvent	Presque toujours
3.	Je me sens content(e) de moi	Presque jamais	Parfois	Souvent	Presque toujours
4.	Je voudrais être aussi heureux (heureuse) que les autres	Presque jamais	Parfois	Souvent	Presque toujours
5.	J'ai un sentiment d'échec	Presque jamais	Parfois	Souvent	Presque toujours
6.	Je me sens reposé(e)	Presque jamais	Parfois	Souvent	Presque toujours
7.	J'ai tout mon sang-froid	Presque jamais	Parfois	Souvent	Presque toujours
8.	J'ai l'impression que les difficultés s'accumulent à un tel point que je ne peux plus les surmonter	Presque jamais	Parfois	Souvent	Presque toujours
9.	Je m'inquière à propos de choses sans importance	Presque jamais	Parfois	Souvent	Presque toujours
10.	Je me sens heureux (heureuse)	Presque jamais	Parfois	Souvent	Presque toujours
11.	J'ai des pensées qui me perturbent	Presque jamais	Parfois	Souvent	Presque toujours
12.	Je manque de confiance en moi	Presque jamais	Parfois	Souvent	Presque toujours
13.	Je me sens sans inquiètude, en sécurité, en sureté	Presque jamais	Parfois	Souvent	Presque toujours
14.	Je prends facilement des décisions	Presque jamais	Parfois	Souvent	Presque toujours
15.	Je me sens incompétent(e), pas à la hauteur	Presque jamais	Parfois	Souvent	Presque toujours
16.	Je suis satisfait(e)	Presque jamais	Parfois	Souvent	Presque toujours
17.	Des idées sans importance trottant dans ma tête me dérangent	Presque jamais	Parfois	Souvent	Presque toujours
18.	Je prends les déceptions tellement à cœur que je les oublie difficilement	Presque jamais	Parfois	Souvent	Presque toujours
19.	Je suis une personne posée, solide, stable	Presque jamais	Parfois	Souvent	Presque toujours
20.	Je deviens tendu(e) et agité(e) quand je réfléchis à mes soucis	Presque jamais	Parfois	Souvent	Presque toujours

Validations : Spielberger, C. D., Gorsuch, R. L., Lushene, R., Vagg, P. R., & Jacobs, G. A. (1983). Manual for the State-Trait Anxiety Inventory (Form Y). Palo Alto, CA: Consulting Psychologists Press.
Spielberger, C. D., & Bruchon-Schweitzer, M. (1993). STAI-Y : Inventaire d'anxiété état-trait forme Y. Éditions du centre de psychologie appliquée.

Annexe 4 : ECHELLE Literatie HLSEU 16

HLS-EU16 version Française (Version 2020)

Indiquez, sur une échelle de très facile à très difficile, dans quelle mesure il est facile pour vous de...

	très facile	facile	difficile	très difficile
... trouver des informations sur les traitements des maladies qui vous concernent ?	❑	❑	❑	❑
... savoir où obtenir l'aide d'un professionnel quand vous êtes malade ? (Par ex. médecin, infirmier, pharmacien ou psychologue)	❑	❑	❑	❑
... comprendre ce qu'un médecin vous dit ?	❑	❑	❑	❑
... comprendre les consignes de votre médecin ou pharmacien sur la manière de prendre vos médicaments ?	❑	❑	❑	❑
... savoir quand il serait utile d'avoir l'avis d'un autre médecin ?	❑	❑	❑	❑
... utiliser les informations que le médecin vous donne pour prendre des décisions concernant votre maladie ?	❑	❑	❑	❑
... suivre les consignes de votre médecin ou pharmacien ?	❑	❑	❑	❑
... trouver des informations sur comment faire en cas de problèmes psychologiques ? (Par ex. stress, dépression ou anxiété)	❑	❑	❑	❑
... comprendre les mises en gardes concernant l'impact sur la santé de certains comportements comme fumer, ne pas faire assez d'exercice et boire trop ?	❑	❑	❑	❑

Tournez la page s'il vous plaît...

Indiquez, sur une échelle de très facile à très difficile, dans quelle mesure il est facile pour vous de...

	très facile	facile	difficile	très difficile
... comprendre les informations sur les dépistages et examens recommandés ? (Par ex. dépistage du cancer colorectal, test de glycémie)	❑	❑	❑	❑
... évaluer la fiabilité des informations disponibles dans les médias sur ce qui est dangereux pour la santé ? (Par ex. journaux, télévision ou internet)	❑	❑	❑	❑
... savoir comment vous protéger des maladies à partir des informations disponibles dans les médias ? (Par ex. journaux, télévision ou internet)	❑	❑	❑	❑
... vous renseigner sur les activités bénéfiques pour votre santé et votre bien être ? (Par ex. relaxation, exercice physique, yoga)	❑	❑	❑	❑
... comprendre les conseils de votre famille ou de vos amis en matière de santé ?	❑	❑	❑	❑
... comprendre les informations disponibles dans les médias pour être en meilleure santé ?	❑	❑	❑	❑
... identifier quels sont les comportements de votre vie de tous les jours qui ont un impact sur votre santé ?	❑	❑	❑	❑

Fin du questionnaire.

Version courte du « European Health Literacy Survey Questionnaire » (Sørensen et al., 2013), validée en français par Rouquette en 2018 (Rouquette et al., 2018).
Disponible sur : https://reflis.fr/wp-content/uploads/2020/07/HLSEU16-Francais-2020.pdf

Annexe 5 : ECHELLE qualité de vie – EQ-5D-5L
Version française pour le Canada

Pour chaque rubrique, veuillez cocher UNE case, celle qui décrit le mieux votre santé AUJOURD'HUI.

MOBILITÉ

Je n'ai aucun problème pour me déplacer à pied ☐
J'ai des problèmes légers pour me déplacer à pied ☐
J'ai des problèmes modérés pour me déplacer à pied ☐
J'ai des problèmes sévères pour me déplacer à pied ☐
Je suis incapable de me déplacer à pied ☐

AUTONOMIE DE LA PERSONNE

Je n'ai aucun problème pour me laver ou m'habiller tout(e) seul(e) ☐
J'ai des problèmes légers pour me laver ou m'habiller tout(e) seul(e) ☐
J'ai des problèmes modérés pour me laver ou m'habiller tout(e) seul(e) ☐
J'ai des problèmes sévères pour me laver ou m'habiller tout(e) seul(e) ☐
Je suis incapable de me laver ou de m'habiller tout(e) seul(e) ☐

ACTIVITÉS COURANTES *(exemples: travail, études, travaux domestiques, activités familiales ou loisirs)*

Je n'ai aucun problème pour accomplir mes activités courantes ☐
J'ai des problèmes légers pour accomplir mes activités courantes ☐
J'ai des problèmes modérés pour accomplir mes activités courantes ☐
J'ai des problèmes sévères pour accomplir mes activités courantes ☐
Je suis incapable d'accomplir mes activités courantes ☐

DOULEURS / INCONFORT

Je n'ai ni douleur ni inconfort ☐
J'ai des douleurs ou un inconfort léger(ères) ☐
J'ai des douleurs ou un inconfort modéré(es) ☐
J'ai des douleurs ou un inconfort sévère(s) ☐
J'ai des douleurs ou un inconfort extrême(s) ☐

ANXIÉTÉ / DÉPRESSION

Je ne suis ni anxieux(se) ni déprimé(e) ☐
Je suis légèrement anxieux(se) ou déprimé(e) ☐
Je suis modérément anxieux(se) ou déprimé(e) ☐
Je suis sévèrement anxieux(se) ou déprimé(e) ☐
Je suis extrêmement anxieux(se) ou déprimé(e) ☐

2

Canada (French) © 2009 EuroQol Group EQ-5D™ is a trade mark of the EuroQol Group

- Nous aimerions savoir dans quelle mesure votre santé est bonne ou mauvaise AUJOURD'HUI.
- Cette échelle est numérotée de 0 à 100.
- 100 correspond à la meilleure santé que vous puissiez imaginer. 0 correspond à la pire santé que vous puissiez imaginer.
- Veuillez faire un X sur l'échelle afin d'indiquer votre état de santé AUJOURD'HUI.
- Maintenant, veuillez noter dans la case ci-dessous le chiffre que vous avez coché sur l'échelle.

VOTRE SANTÉ AUJOURD'HUI = ☐

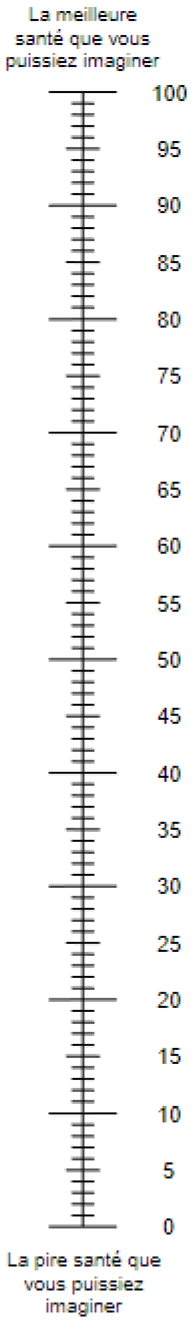

Canada (French) © 2009 EuroQol Group EQ-5D™ is a trade mark of the EuroQol Group

Annexe 6 : QUESTIONNAIRE de WAKE

How would you rate your understanding of your cancer/disease?	Very poor	Poor	Fair	Good	Very good
I understand how big my cancer/tumor is.	Strongly disagree	Disagree	Neutral	Agree	Strongly agree
I understand where my cancer/tumor is located.	Strongly disagree	Disagree	Neutral	Agree	Strongly agree
I understand why my surgeon chose the treatment plan being offered.	Strongly disagree	Disagree	Neutral	Agree	Strongly agree
I feel comfortable with the surgical plan.	Strongly disagree	Disagree	Neutral	Agree	Strongly agree

Version traduite (par le CeDS)

Comment évaluez-vous votre compréhension de votre maladie ?	Très mauvaise	mauvaise	correcte	Bonne	Très bonne
Je comprends la taille de mon cancer	Pas du tout d'accord	Pas d'accord	Sans avis	D'accord	Tout à fait d'accord
Je sais où mon cancer est localisé	Pas du tout d'accord	Pas d'accord	Sans avis	D'accord	Tout à fait d'accord
Je comprends le traitement qui est choisi	Pas du tout d'accord	Pas d'accord	Sans avis	D'accord	Tout à fait d'accord
Je me sens confiant avec le traitement choisi	Pas du tout d'accord	Pas d'accord	Sans avis	D'accord	Tout à fait d'accord

Wake N, Rosenkrantz AB, Huang R, Park KU, Wysock JS, Taneja SS, Huang WC, Sodickson DK, Chandarana H. Patient-specific 3D printed and augmented reality kidney and prostate cancer models: impact on patient education. 3D Print Med. 2019 Feb 19;5(1):4. doi: 10.1186/s41205-019-0041-3. PMID: 30783869; PMCID: PMC6743040.

Annexe 7 : Consentement UroCCR

Projet UroCCR et UroCCR Chain
Version n°5.0 du 13/03/2023

NOTE D'INFORMATION POUR LA CONSTITUTION DE BASES DE DONNEES A VISEE DE RECHERCHE ET D'EVALUATION DES SOINS AVEC COLLECTION VIRTUELLE D'ECHANTILLONS BIOLOGIQUES SUR LE CANCER DU REIN : UROCCR ET UROCCR-CHAIN

Gestionnaire du Réseau UroCCR : **CHU de Bordeaux**
Coordonnateur : **Pr Jean-Christophe BERNHARD**

Madame, Monsieur,

Votre médecin spécialiste et le service auquel il appartient participent à un projet multicentrique national soutenu par l'Institut National du Cancer (INCa). Ce projet, intitulé UroCCR, consiste en la création et le déploiement d'une base de données dédiée à la recherche multidisciplinaire sur le cancer du rein, associée à une banque de ressources biologiques virtuelle.

Lors de votre prise en charge médicale, des prélèvements biologiques (sang, urines, tissus…) peuvent être effectués afin de permettre un diagnostic, la surveillance de votre maladie et son traitement. Cette prise en charge peut également nécessiter le recueil de données médicales. A l'issue de leur utilisation à des fins médicales, ces ressources biologiques et ces données sont susceptibles de présenter un intérêt pour la recherche scientifique en lien avec votre pathologie, réalisée sous contrôle de la pertinence scientifique par un comité d'experts.

Les médecins des différentes spécialités impliquées (Chirurgie Urologique, Oncologie médicale, Imagerie médicale et anatomo-pathologie notamment), pourront ainsi collecter l'ensemble des renseignements cliniques et biologiques en rapport avec votre maladie et sa prise en charge.

Dans le cadre d'UroCCR, le Gestionnaire pourra communiquer des informations personnelles aux agences réglementaires ou à ses partenaires de recherche. Ces personnes, sociétés et agences peuvent être situées en France, dans d'autres pays de l'Espace Economique Européen (EEE), aux États-Unis et dans d'autres pays à l'extérieur de l'EEE. Il est possible que certains pays hors de l'EEE n'offrent pas le même niveau de protection de la vie privée qu'en France. Dans ce cas, le Gestionnaire maintiendra toutefois la confidentialité de toutes les informations personnelles qu'il échangera dans les limites de la loi. Le Gestionnaire adoptera les mesures contractuelles appropriées relatives à la protection et au transfert des données, pour s'assurer que les destinataires pertinents en dehors de l'EEE fournissent un niveau adéquat de protection concernant vos informations personnelles et conformément à la loi.

En parallèle, des données du Système National des Données de Santé (« SNDS », c'est-à-dire les données de l'Assurance Maladie) permet d'obtenir une meilleure compréhension de vos pathologies dans la mesure où elles permettent d'avoir un suivi plus complet de chaque patient. Dans ce cadre, le CHU de Bordeaux a également décidé de mettre en œuvre, en collaboration avec la société Clinityx, un second projet « UroCCR-Chain », afin d'intégrer ces données pour permettre de réaliser des analyses.

Il est important que vous lisiez attentivement ces pages qui vous apporteront des informations sur l'utilité d'un tel recueil de données ainsi que sur ses modalités pratiques. N'hésitez pas à nous poser toutes les questions que vous jugerez utiles.

Pour rappel, UroCCR et UroCCR-Chain sont deux projets, qui consistent en la mise en place d'une Base de Données pour le premier et d'un Entrepôt de données de santé (EDS) pour le second, dont l'objectif commun est de mettre en place de nombreuses études ultérieurement.

<u>Les informations relatives aux différents projets menés seront disponibles sur www.uroccr.fr</u>.

Vous pouvez à tout moment vous opposer à ce que vos échantillons et données soient utilisés pour tout ou partie des projets référencés sur www.uroccr.fr sans avoir de justification à fournir.

Projet UroCCR et UroCCR Chain
Version n°5.0 du 13/03/2023

<table>
<tr><th></th><th>UroCCR</th><th>UroCCR-Chain</th></tr>
<tr><td>Personnes en charge du projet (Responsable de traitement ou coresponsables de traitement)</td><td>Le CHU de Bordeaux dont la Direction Générale est située 12 rue Dubernat 33400 Talence France.</td><td>Le CHU de Bordeaux et Clinityx (société spécialisée dans l'analyse de données de l'Assurance Maladie et la mise en place d'entrepôts de données de santé) dont le siège social est situé 137 rue d'Aguesseau 92160 Boulogne Billancourt</td></tr>
<tr><td>Finalité (Raison de la mise en place des projets)</td><td>Les objectifs d'UroCCR sont :
- permettre la réalisation d'études épidémiologiques, observationnelles ou translationnelles, à échelon national ou international, dans le domaine du cancer du rein, liées à :
- l'épidémiologie descriptive du cancer du rein ;
- la pharmaco-épidémiologie et l'observation des traitements ;
- la qualité de vie et les conséquences personnelles, familiales, profession-nelles et sociales du cancer du rein ;
- la recherche de biomarqueurs diagnostiques et pronostiques ;
- la recherche de marqueurs prédicteurs de l'évolution de la maladie ;
- l'évaluation des pratiques de soins et des techniques de traitement.</td><td>L'entrepôt de données de santé « UroCCR-Chain » a les mêmes finalités qu'UroCCR avec des données enrichies de l'assurance maladie afin d'obtenir un suivi complet de la pathologie et de disposer d'information médico-économiques (coûts de prise en charge).</td></tr>
<tr><td>Base légale (raison juridique de la mise en place de ce projet)</td><td>Le recueil/traitement de ces données sera fait à des fins de recherche scientifique et trouve son fondement dans l'intérêt public des missions du CHU de Bordeaux.</td><td>Le traitement mis en œuvre par les coresponsables de traitement (CHU de Bordeaux et Clinityx) est nécessaire aux fins des intérêts légitimes qu'ils poursuivent.</td></tr>
<tr><td>Destinataires des données à caractère personnel</td><td>Etablissements participants au réseau UroCCR et autres partenaires de recherche dans le cadre des projets ancillaires.</td><td>Le CHU de Bordeaux et Clinityx uniquement.</td></tr>
<tr><td rowspan="2">Informations concernées (Données personnelles)</td><td>Données de santé vous concernant, données relatives au phénotype africain et données génétiques issues de votre dossier médical.</td><td>Les mêmes données de santé que pour UroCCR enrichies des données de l'Assurance Maladie (consommation de soins, consul-tations, traitements, hospitalisations, statut vital…).</td></tr>
<tr><td colspan="2">Afin d'assurer la confidentialité de vos informations à caractère personnel, ni votre nom ni aucune autre information qui permettraient de vous identifier directement ne seront saisis dans un dossier ou sur un échantillon que le médecin du projet fournira au Gestionnaire ou aux représentants autorisés du Gestionnaire. Vous serez uniquement identifié(e) par un code et vos initiales. Le code est utilisé pour que le médecin du projet puisse vous identifier si nécessaire. Ces données seront enregistrées sur des serveurs informatiques sécurisés.</td></tr>
<tr><td>Durée de conservation</td><td>15 ans après le dernier suivi du patient.</td><td>10 ans à compter de la mise en place du projet.</td></tr>
</table>

Projet UroCCR et UroCCR Chain
Version n°5.0 du 13/03/2023

Coordonnées du Délégué à la protection des données personnelles	Le Délégué à la protection des données personnelles du CHU de Bordeaux : mesdonneespersonnelles@chu-bordeaux.fr	
Vos droits (loi relative à l'informatique, aux fichiers et aux libertés n° 78-17 du 6 janvier 1978 relative à l'informatique, aux fichiers et aux libertés modifiée par la loi n° 2018-493 du 20 juin 2018 relative à la protection des données personnelles ; Règlement général sur la protection des données UE 2016/679)	Vous disposez des droits suivants sur vos données collectées et générées dans le cadre de votre participation :<br>- Droit d'accès aux données,<br>- Droit de rectification des données erronées,<br>- Droit d'effacement des données en cas de traitement illicite,<br>- Droit de portabilité vous permettant d'obtenir les données que vous avez-vous-même fournies,<br>- Droit de limitation du traitement des données notamment si celui-ci venait à être remis en cause.	Vous disposez des droits suivants sur vos données collectées et générées dans le cadre de votre participation :<br>- Droit d'accès aux données,<br>- Droit de rectification des données erronées,<br>- Droit d'effacement des données en cas de traitement illicite,<br>- Droit de limitation du traitement des données notamment si celui-ci venait à être remis en cause.
	Vous disposez également d'un droit d'opposition au traitement de vos données. Dès lors, aucune nouvelle donnée à caractère personnel vous concernant ne sera collectée. L'exercice de ce droit empêche tout traitement ultérieur des données vous concernant et vous ne pourrez plus poursuivre votre participation à cette recherche. Toutefois, toute information déjà collectée au préalable sera conservée et continuera d'être utilisée dans le cadre de ces recherches.<br><br>Vous pouvez exercer ces droits en le demandant par écrit auprès du médecin qui vous suit dans le cadre de ces recherches qui transmettra la demande au CHU de Bordeaux. Le CHU de Bordeaux répondra à vos demandes conformément à ses obligations légales et réglementaires.<br><br>Vous pouvez également accéder directement ou par l'intermédiaire du médecin de votre choix à l'ensemble de vos données médicales en application des dispositions de l'article L1111-7 du code de la santé publique. Ce droit s'exerce auprès du médecin qui vous suit dans le cadre de ses recherches et qui connaît votre identité.	

En cas d'insatisfaction, vous pouvez déposer une réclamation auprès de l'autorité de surveillance de la protection des données, la Commission Nationale de l'Informatique et des Libertés (CNIL) à l'adresse : https://www.cnil.fr/fr/webform/nous-contacter ou https://www.cnil.fr/fr/plaintes/.

Les résultats des projets menés sur UroCCR et UroCCR Chain pourront donner lieu à des innovations brevetées ou protégées par le droit de la propriété intellectuelle, et à des publications scientifiques.

Lorsque ces projets seront terminés, les résultats globaux seront accessibles sur le site www.uroccr.fr ou par votre médecin dès que ceux-ci seront disponibles et si vous le souhaitez.

Projet UroCCR et UroCCR Chain
Version n°5.0 du 13/03/2023

NOTE D'INFORMATION POUR LA CONSTITUTION DE BASES DE DONNEES A VISEE DE RECHERCHE ET D'EVALUATION DES SOINS AVEC COLLECTION VIRTUELLE D'ECHANTILLONS BIOLOGIQUES SUR LE CANCER DU REIN : **UROCCR ET UROCCR-CHAIN**

En remplissant ce document vous certifiez avoir pris connaissance de la note d'information jointe et avoir pu poser toutes les questions utiles à votre médecin. Merci de nous faire part de votre décision en complétant et en signant le formulaire joint ci-dessous.

Identification

Je soussigné(e)
Nom de famille (patronymique) : ....

Prénom : ....

Né(e) le : |__|__| (jj) |__|__| (mm) |__|__|__|__| (aaaa) Ville de naissance : ....

Pour les majeurs sous tutelle *(les majeurs sous curatelle ou sous sauvegarde de justice ne peuvent participer à ces activités)**, le représentant légal doit compléter les mentions ci-dessous*** :

Le représentant légal : ❑ Mme ❑ M.

Nom de famille : ....

Prénom : .... Né(e) le : |__|__| (jj) |__|__| (mm) |__|__|__|__| (aaaa)

Merci d'indiquer votre décision en cochant les cases correspondant à votre choix

Compte tenu de ces informations :

	**J'accepte :**	**Je refuse :**
Les échantillons biologiques et les informations médicales disponibles au décours de ma prise en charge peuvent être collectés, conservés et utilisés par mon établissement de soins participant à UroCCR ou transférés à l'usage d'autres équipes scientifiques dans le cadre du Projet UroCCR, jusqu'à leur épuisement (utilisation totale). La présentation des projets ancillaires est accessible sur le site www.uroccr.fr. L'affichage des projets de recherche sur cette page Internet vaut information, sans lettre d'information individuelle complémentaire.	❒	❒
Ces recherches non interventionnelles pourront comprendre l'examen de mes caractéristiques génétiques sans finalité identifiante. Elles ne pourront pas modifier ma prise en charge médicale et la manière dont me sont dispensés les soins.	❒	❒
J'accepte de participer à UroCCR-Chain. La présentation des projets ancillaires est accessible sur le site www.uroccr.fr.	❒	❒

Ce formulaire, ainsi que toutes les informations personnelles me concernant resteront strictement confidentiels, sont et resteront couverts par le secret professionnel et médical, ainsi que par le respect dû à ma vie privée. En vertu de la loi « Informatique et Libertés » du 6 janvier 1978 modifiée (notamment par la loi n° 2018-493 du 20 juin 2018 relative à la protection des données personnelles) et au règlement général sur la protection des données (règlement UE 2016/679), je dispose d'un droit d'accès, de rectification, de suppression, de limitation du traitement, à la portabilité des données (non applicable pour UroCCR-Chain), d'opposition et de retrait.
J'ai pris connaissance que :
- le projet UroCCR a reçu l'avis favorable du Comité Consultatif sur le Traitement de l'Information en matière de Recherche dans le domaine de la Santé (CCTIRS) le 24/10/2012 et l'autorisation de la Commission nationale de l'informatique et des libertés (CNIL) le 12/04/2013 (Décision DR-2013-206) et le 15/12/2016 (Décision DR-2016-485).
- l'entrepôt de données de santé UroCCR-Chain a reçu l'autorisation de la CNIL dans sa délibération n°2022-091 du 08/09/2022

Je suis libre de revenir sur ma décision en le signifiant par e-mail via www.uroccr.fr ou par téléphone au 05 57 82 23 94

Fait à :	Fait à : ....<br>Nom du médecin:	Fait à :..
Le : |__|__| / |__|__| / |__|__|__|__|	Le : |__|__| / |__|__| / |__|__|__|__|	Le : |__|__| / |__|__| / |__|__|__|__|
Signature du Patient	Signature du Médecin	Signature du représentant du Patient
